# Modulation of defensive reactivity by *GLRB* allelic variation: converging evidence from an intermediate phenotype approach

**DOI:** 10.1038/tp.2017.186

**Published:** 2017-09-05

**Authors:** U Lueken, M Kuhn, Y Yang, B Straube, T Kircher, H-U Wittchen, B Pfleiderer, V Arolt, A Wittmann, A Ströhle, H Weber, A Reif, K Domschke, J Deckert, T B Lonsdorf

**Affiliations:** 1 Center of Mental Health, Department of Psychiatry, Psychosomatics, and Psychotherapy, University Hospital of Würzburg, Würzburg, Germany; 2 Department of Systems Neuroscience, University Medical Center Hamburg-Eppendorf, Hamburg, Germany; 3 Department of Psychiatry and Psychotherapy, Phillips-University Marburg, Marburg, Germany; 4 Department of Psychology, Institute of Clinical Psychology and Psychotherapy, Technische Universität Dresden, Dresden, Germany; 5 Department of Clinical Radiology, University Hospital Münster, Münster, Germany; 6 Department of Psychiatry, University Hospital Münster, Münster, Germany; 7 Department of Psychiatry and Psychotherapy, Charité - University Medicine Berlin, Berlin, Germany; 8 Department of Psychiatry, Psychosomatic Medicine and Psychotherapy, University Hospital Frankfurt, Frankfurt am Main, Germany; 9 Department of Psychiatry and Psychotherapy, Medical Center, Faculty of Medicine, University of Freiburg, Freiburg, Germany

## Abstract

Representing a phylogenetically old and very basic mechanism of inhibitory neurotransmission, glycine receptors have been implicated in the modulation of behavioral components underlying defensive responding toward threat. As one of the first findings being confirmed by genome-wide association studies for the phenotype of panic disorder and agoraphobia, allelic variation in a gene coding for the glycine receptor beta subunit (*GLRB*) has recently been associated with increased neural fear network activation and enhanced acoustic startle reflexes. On the basis of two independent healthy control samples, we here aimed to further explore the functional significance of the *GLRB* genotype (rs7688285) by employing an intermediate phenotype approach. We focused on the phenotype of defensive system reactivity across the levels of brain function, structure, and physiology. Converging evidence across both samples was found for increased neurofunctional activation in the (anterior) insular cortex in *GLRB* risk allele carriers and altered fear conditioning as a function of genotype. The robustness of *GLRB* effects is demonstrated by consistent findings across different experimental fear conditioning paradigms and recording sites. Altogether, findings provide translational evidence for glycine neurotransmission as a modulator of the brain’s evolutionary old dynamic defensive system and provide further support for a strong, biologically plausible candidate intermediate phenotype of defensive reactivity. As such, glycine-dependent neurotransmission may open up new avenues for mechanistic research on the etiopathogenesis of fear and anxiety disorders.

## Introduction

Glycine receptors, including its beta receptor subunit (*GLRB*), play a major role for inhibitory neurotransmission. Recently, a genome-wide association of *GLRB* with categorical (panic disorder (PD)) and dimensional (agoraphobia (AG)) forms of fear and anxiety has been reported,^1^ in particular for rs7688285 which was associated with *GLRB* expression changes in post mortem tissue and reporter gene assays. Increased neural fear network activation during fear conditioning and increased acoustic startle reflexes were observed in risk allele carriers, thus qualifying as a potential intermediate phenotype of defensive system reactivity across different levels of analyses, corresponding to the Research Domain Criteria (RDoC) approach.^2^

In light of the recent ‘replication crisis’ in psychology and neuroscience,^3, 4^ such promising findings need to be followed up. Direct replication attempts (for example, replication of genome-wide association studies (GWAS) findings in an independent sample as performed in the initial report) provide information about the reproducibility of a specific phenomenon, but not necessarily about the theoretical construct.^3^ Hence, direct replications need to form a synergy with conceptual replications targeting the same construct with divergent methodological approaches and paradigms.^3^ Here we focus on ‘defensive reactivity’ on a neurobiological level of analysis aiming to conceptually replicate the previous report^1^ on fear conditioning and startle responding.

Defensive behaviors are part of an evolutionary conserved dynamic defense cascade that depends upon the proximity of threat, as described in the predator imminence model.^5, 6^ The defensive system encompasses phylogenetically older adaptive behaviors (startle reflex; fight, flight, freeze responses) to higher-level integrated cognitive-affective coping systems.^7^ The underlying neural circuit is represented by the interplay of diverse cortical structures such as the medial prefrontal cortex, anterior cingulate cortex (ACC), insular cortex, hippocampus, and amygdala that dynamically interact with midbrain structures (periaqueductal grey) mediating hard-wired defensive reflexes under proximal threat.^5, 8, 9, 10^

Phylogenetically old defensive responses are modulated by individual learning experiences. Fear conditioning enables the organism to avoid future threats in that important information (conditioned stimuli (CS)) signaling a potential threat (unconditioned stimulus (US)) elicits defensive reactions (conditioned fear response). Fear conditioning is considered to be involved in the pathogenesis of pathological anxiety and serves as experimental model for the development of anxiety disorders. On a neural level, fear conditioning involves multiple areas associated with defensive responding such as the (pre-) motor cortex, medial prefrontal cortex/ACC, anterior insula, amygdala, hippocampus and thalamus.^11, 12^

Human^13, 14^ and animal studies^15^ evidence that genetic factors represent a significant source of individual variation in (aversive) emotional-associative learning. At least one third of the variance in human fear conditioning^14^ and the risk to develop anxiety disorders^16^ has been attributed to genetic factors. The overall startle magnitude in humans has been shown to be heritable (for example, 59–61% in^17^ or 37–52% in^18^). Studies on the heritability of task-related brain activity suggest moderate heritability of brain activation in general^19, 20^ and amygdala activation (to faces) appears to be a temporally stable and trait-like measure.^21^ This evidence presents fear conditioning and affective startle paradigms as prime laboratory models for investigating genetic influences on neurobiological mechanisms contributing to defensive responding, fear and anxiety.

On the basis of previous findings^1^ and focusing on rs7688285 which phenotypically showed the most robust impact, we aimed to conduct a conceptual replication by studying defensive reactivity on the level of brain function, structure, and physiology. We expected *GLRB* risk allele carriers to (a) exhibit increased fear network activation during two different fear conditioning tasks in regions related to the defensive system’s neuro-architecture such as the brainstem, thalamus, amygdala, hippocampus, insula, medial prefrontal cortex/ACC, and (pre-) motor cortex with different effects for the early vs late acquisition (see^1^), (b) show brain morphometric alterations in these areas of interest that could underlie observed functional changes, and (c) show impaired startle habituation as a subsequent behavioral outflow of enhanced defensive system reactivity.

## Materials and methods

### Sample characteristics

*Sample 1*: Sample 1 from Hamburg was recruited from a healthy subject pool within a collaborative research center (CRC TRR-58, first and second funding period, see Supplementary Table S1 for sample descriptives) and partly included in the GWAS analyses reported in.^1^ However, this sample originated from a different project within the CRC TRR-58, using a complex cue and context conditioning procedure. For subjects already included in the previous GWAS analysis we present new data on associations between *GLRB* genetic variants and brain function (*n*=48) and morphology (*n*=416). In addition, we present data of a further sample on *GLRB* dependent variation of startle habituation (*n*=105, of which seven subjects were included in the previous GWAS). Subjects were screened for psychiatric conditions via the mini-international neuropsychiatric interview,^22^ and excluded if screening for any lifetime mental disorder was positive. They provided written informed consent and protocols were approved by the General Medical Council of the State of Hamburg.

*Sample 2:* This healthy control group sample was recruited within the national research network PANIC-NET^23^ (Supplementary Table S1) and not part of the previous report. Four centers (Aachen, Berlin-Charité, Dresden, Münster) participated in a longitudinal functional magnetic resonance imaging (fMRI) study that aimed to detect neuroplastic changes following exposure-based cognitive-behavioral therapy in PD with AG. Quality controlled fMRI data from *n*=60 controls^24^ were included from whom genotype information on rs7688285 was available in *n*=38 subjects. Subjects were free of lifetime mental disorders as evidenced by the Composite International Diagnostic Interview (CAPI-WHO-CIDI; DIAX-CIDI version).^25^ After complete description of the study, subjects provided informed written consent. The study had been approved by the ethics committees of all participating centers.

### Genotyping

Genotyping based on blood samples was performed with Sequenom's MassArray system (Sequenom, San Diego, CA, USA), as described previously.^1^ Focusing on rs7688285 as main SNP of interest, the Risk group was defined as carrying at least one risk (A) allele (G/A). Complying with the previous report,^1^ rs7688285 and three further *GLRB* variants (rs17035763: G/A with A allele as risk allele, rs191260602: A/G with G allele as risk allele, and rs78726293: T/A with A allele as risk allele) were used for the Combined *GLRB* Risk group analyses (see Supplementary Information). Combined Risk group status was defined as carrying at least one risk allele out of the four SNPs.

### Data acquisition and analysis pathways

#### Sample 1

The fMRI assessment employed a combined cue and context fear conditioning paradigm (for details see^26, 27, 28^). Subjects viewed a sequence of three different rooms (45 s each) as context CSs, which were assigned counterbalanced to a cue conditioning (predictable: p), a context conditioning (unpredictable: u) or a safe condition (s). During fixed time windows, three different symbols that served as cue CSs (5 s) were superimposed on the assigned room. A black screen with a white fixation cross served as inter-trial interval for 6–8 s (mean 7 s). An individually adjusted electro-tactile stimulus served as US. To mirror experimental designs reported in^1^ and in sample 2, only responses based on cued, not context fear were considered. Subjective ratings and mean skin conductance reactions across all cue onsets were analyzed by an analysis of variance (ANOVA) with CS-type as within-subject variable and genotype as between-subject variable across all blocks. Details on data acquisition, post-processing, and response definition for skin conductance reaction have been reported previously.^26, 27, 28^ Data of seven subjects were excluded because their proportion of zero or missing responses exceeded a threshold of 66% during the full fear acquisition phase. An α level of *P*<0.05 was considered significant and Greenhouse–Geisser correction was applied if necessary.

Functional and structural magnetic resonance imaging data were acquired on a 3 T MRI scanner (MAGNETOM Trio, Siemens, Erlangen, Germany). For fMRI, 38 continuous axial slices (2 mm thick, 1 mm gap) were acquired using a T2*-sensitive gradient echo-planar imaging (EPI) sequence (repetition time (TR): 2.34 s; echo time (TE): 26 ms; flip angle: 80° field of view (FOV): 220 × 220 mm, 2 × 2 mm in-plane resolution) in three sessions (229 volumes/~9 min each) using a 32-channel head coil (generalized auto-calibrating partially parallel acquisitions, GRAPPA-factor 2). To account for T1 equilibrium effects, the first four volumes of each time series were discarded. fMRI preprocessing included realignment, unwarping, co-registration and normalization (using DARTEL^29^) with SPM8 (http://www.fil.ion.ucl.ac.uk/spm/) running on MATLAB2013a (The MathWorks, Natick, MA, USA) smoothed with a 6 mm full-width at half maximum (FWHM) isotropic Gaussian kernel. For first-level analyses, data were analyzed within the framework of the general lineal model for differences in general and differential cued fear responses (that is, CS+ & CS− CS+ vs CS−). Linear models for each of the three sessions included block regressors for three contexts (PCXT, UCXT, SCXT) and one block regressor for the rating phase. Event-regressors were included for three cue- (PCue, UCue, SCue) and US-onsets and convolved with the canonical hemodynamic response function. Parameter estimates (β−) images were calculated and linearly combined for contrasts for main effect responses (CS+ & CS−), as well as differential responses (CS+ vs CS−). As the previous study^1^ reported findings particularly during early acquisition, we computed first-level contrasts for early acquisition (session one only), late acquisition (session two and three), as well as full acquisition (all sessions). Second-level group analyses were performed using general lineal model analyses for early, late and full acquisition by two-sample *t*-test designs. Contrasts of interest included the main effect of group (Risk: AG, GG carriers vs No-Risk: GG carriers) and the interaction effect of group * CS (CS+ vs CS−). Because of significant group differences in mean age (see Supplementary Table S1), second-level analyses included age as regressor of no interest. The following regions of interest (ROI) were used based on the automatic anatomical labeling atlas^30^ and the Tailarach Daemon lobes as implemented in the wfu pickatlas:^31^ brainstem (medulla, pons, and midbrain), thalamus, amygdala, hippocampus, insula, ACC, medial prefrontal cortex (superior medial frontal gyrus, medial orbitofrontal gyrus), supplementary motor areal and precentral gyrus. Small volume corrections were based on a cluster forming threshold of *P*<0.001 and a family-wise error threshold of *P*<0.05. Beta values from significant peak voxels were extracted for bar graph visualization.

Details of structural (s) MRI for brain morphometry have been reported previously.^27^ High-resolution T1-weighted images (1 × 1 × 1 mm) were acquired using a magnetization prepared rapid gradient echo sequence (MPRAGE) using a 12- or a 32-channel head coil (coil number was included as covariate). Grey matter differences were analyzed using the voxel-based morphometry (VBM) toolbox (VBM8, version 435, www.http://dbm.neuro.uni-jena.de/ vbm/) as provided for SPM8. Default settings included a ‘non-linear only’ modulation of the grey matter (GM). Pre-processed images were smoothed with a Gaussian kernel of 8 mm FWHM. Analysis of the covariance between the images identified no images as outliers (mean−2 × s.d.). Correction of multiple comparisons followed the regions of interest approach of the fMRI analyses above. An additional exploratory whole-brain analysis was computed using an uncorrected threshold of *P*<0.001 and a minimum cluster size of k>=15. Where appropriate, covariates were added if groups differed in demographic or personality properties (for example, ASI sum score).

Startle reactions were measured by recording electromyographic activity over the orbicularis oculi muscle beneath the left eye using miniature Ag/AgCl electrodes. A 50 ms burst of 95 dB(A) white noise with an instantaneous rise-time was presented binaurally via headphones (Sennheiser, Wedemark, Germany) as startle-eliciting stimulus. In correspondence with,^1^ startle habituation was assessed for 12 blink responses during inter-trial intervals interspersed during passive viewing of emotional pictures in an affective startle modulation paradigm. Although the task was conceptually similar to the paradigm employed by the previous report, subtle differences with respect to timings, experimental breaks, or selected stimulus material exist. An ANOVA testing for a direct interaction effect of the within- (time block) and the between-subject (genotype) factors was computed. All behavioral and psychophysiological data of the sample were analyzed using SPSS 22 (IBM, Armonk, NY, USA) for Windows.

#### Sample 2

A differential fear conditioning and extinction task was employed that has been described elsewhere.^32^ It consisted of three phases (familiarization (F) with 16 trials; acquisition (A) with 32 trials and extinction (E) with 16 trials of each CS (colored geometrical forms); presentation time: 2000 ms with a variable inter-trial interval of 4.785 to 7.250 s) and an aversive tone (white noise; 100 ms) as US between 70 and 105 dB. In the acquisition, the US was pseudorandomly paired with one of the CSs (counterbalanced between subjects; partial reinforcement rate of 50%), resulting in equal proportions of CS+paired and CS+unpaired trials of which only CS+unpaired trials were used to avoid confounding effects between CS+ and US processing. Structural data with sufficient high-resolution were not available for sample 2. After each phase, valence and arousal ratings using the Self-Assessment Manikin^33^ for CSs were obtained using a five-point Likert Scale (for valence: 1=‘very unpleasant’ to 5=‘very pleasant’ and for arousal: 1=‘not arousing’ to 5= ‘very arousing’). Two subjects that rated the US aversiveness below 4 (on a 10-point Likert scale) were excluded from analyses. Because of technical problems ratings of one subject were missing. Task duration was 16:49 min. Stimuli were presented by MR-compatible LCD goggles or back projection systems and standard headphones using Presentation 11 (Neurobehavioral Systems, http://www.neurobs.com). Ratings were analyzed using a three-factorial analysis of covariance with the between-subjects factor ‘risk group’, the within-subjects factors ‘phase’ and ‘CS’ and Beck Depression inventory II (BDI II)^34^ scores as covariate, with *P*<0.05 serving as the statistical threshold. If sphericity assumptions were violated, Greenhouse-Geisser correction was applied.

Images were acquired using 3 T Philips Achieva (Aachen and Münster), 3 T Siemens Trio (Dresden), and 3 T General Electric Healthcare (Berlin) scanners. Five-hundred and five axial functional images (EPI, matrix 64 × 64, 30 slices interleaved, FOV=230, voxel size=3.6 × 3.6 × 3.8 mm, TE=30 ms, TR=2 s), covering the whole brain were recorded. Images were analyzed using SPM5 implemented in MATLAB 6.5. The first five volumes were discarded to minimize T1 saturation effects. Data were filtered to 1/128 Hz to remove low-frequency noise. Functional images were slice-time corrected, temporally and spatially aligned and normalized into standard stereotactic space (2 × 2 × 2 mm). To account for differences in intrinsic smoothness between scanners, an iterative smoothness equalization procedure^35^ was performed using a target smoothness of 12 mm FWHM Gaussian isotropic kernel (~ 8 mm FWHM in a normal smoothing procedure). A detailed description of measures for quality control is given in.^32^ At first level, realignment parameters were included as regressors of no interest. The BOLD response for each event type (CS+paired, CS+unpaired, CS-, US) and phase (F, A, E) was modeled by the canonical hemodynamic response function within the framework of the general linear model. Parameter estimates (ß-) and t-statistic images were calculated. At the second level, a group analysis was performed by entering contrast images into a flexible factorial analysis. FMRI center variables and the BDI II were introduced as covariates of no interest. Contrasts of interest included the main effect of group (Risk: AG, GG carriers vs No-Risk: GG carriers) during acquisition and - in order to test for the specificity of effects for fear learning - extinction, as well as the interaction effect of group × CS (CS+ unpaired vs CS−) for each experimental phase. Corresponding to,^1^ we further split acquisition as well as extinction into early vs late parts. Two-sample *t*-tests were used in order to localize the direction of effects. Identical ROI analyses were performed as in sample 1. For exploratory whole-brain analyses and in line with previous analyses of this dataset,^24, 32, 36^ a Monte-Carlo simulation was conducted to establish an appropriate voxel contiguity threshold.^37^ Assuming an individual voxel type I error of *P*<0.005, a cluster extent of 142 contiguous resampled voxels was indicated as sufficient to correct for multiple voxel comparisons at *P*<0.05. Beta values from significant peak voxels were extracted for bar graph visualization.

## Results

### *GLRB* modulation of fear network activation

#### Main effect of *GLRB* group

*Sample 1*. While cue-related acquisition of fear during fMRI was indicated by a significant main effect of CS-type for subjective ratings and skin conductance reactions, no main or interaction effect with *GLRB* risk genotype was observed (Supplementary Table S2 and Supplementary Figure S1).

**Figure 1:**
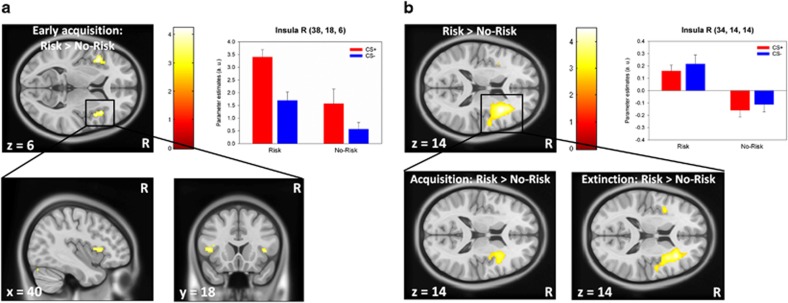
Main effect of *GLRB* Risk group during fear conditioning in sample 1 (**a**; *n*=48) and sample 2 (**b**; *n*=38). In both samples, the Risk groups showed enhanced overall activation of the right anterior insula. While this effect was restricted to the early acquisition in sample 1, Risk allele carriers from sample 2 showed sustained activation throughout the acquisition (early and late) and extinction. Risk group status was defined as carrying at least one risk allele (A allele). CS+, stimulus that was followed by the unconditioned stimulus; CS−, stimulus that was never followed by the unconditioned stimulus; R, right. Threshold at *P*=0.005 for illustrative purposes.

On a neural level, a main effect was observed for the *GLRB* Risk group during early fear acquisition in the left (anterior) insula / frontal operculum area (*x*, *y*, *z*: −42, 14, 6; *t*=4.20, *P*=0.027; Figure 1) and mid-brain (*x*, *y*, *z*: −8, −20, −16; *t*=4.19, *P*=0.030). During the full course of acquisition, increased activation was observed for *GLRB* risk carrier in right insula (*x*, *y*, *z*: 34, −18, 20; *t*=3.90, *P*=0.049) and left amygdala (*x*, *y*, *z*: −16, −2, −12; *t*=3.92, *P*=0.007). Similar findings were obtained when using the Combined Risk group definition as in the previous report^1^ (see Supplementary Table S6 for sample characteristics and S7 for fMRI results).

*Sample 2.* While subjective ratings indicated successful fear conditioning as shown by a significant time × CS interaction effect for valence and a strong, yet insignificant trend for arousal, no main or interaction effects with *GLRB* were observed (Supplementary Table S3 and Supplementary Figure S2).

On a neural level, risk allele carriers showed enhanced activation in the right anterior insula compared with the no-risk group. This effect was equally driven by acquisition and extinction phases and was irrespective of stimulus type (Table 1, Figure 1). Similar findings were obtained for the Combined Risk group definition (Supplementary Tables S6 and S10).

#### Interaction effect *GLRB* group × CS

**Figure 2:**
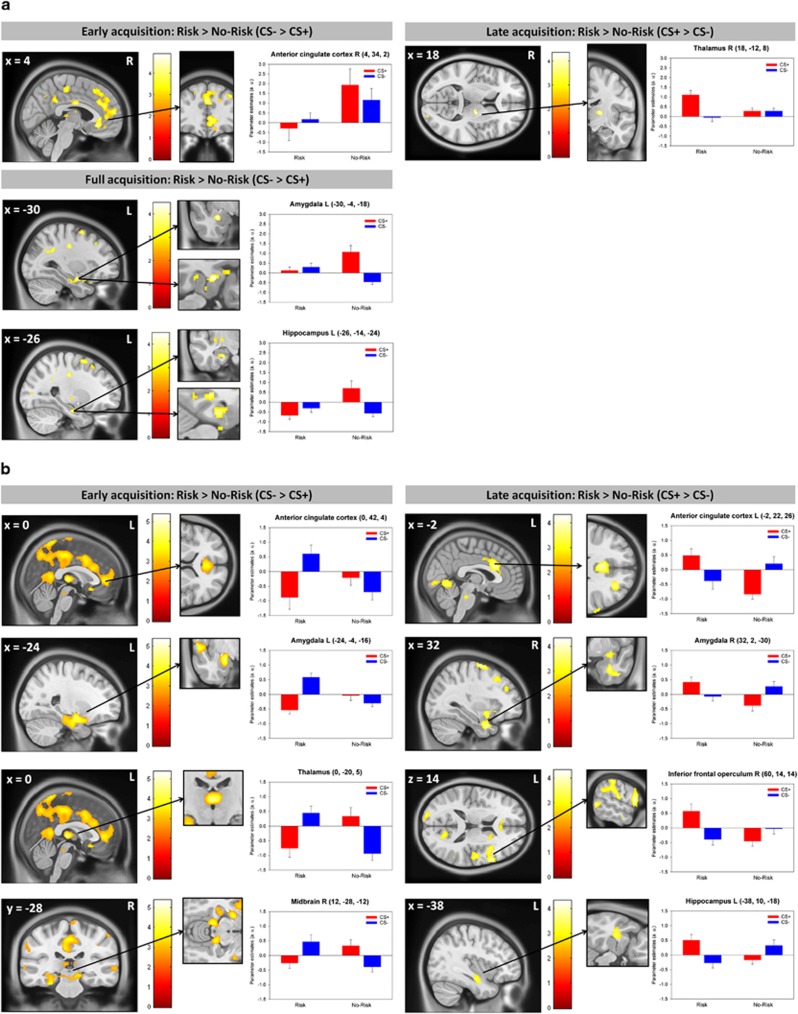
Interaction effect of *GLRB* Risk group and conditioned stimuli during fear conditioning in sample 1 (**a**; *n*=48) and sample 2 (**b**; *n*=38). During early acquisition (left), Risk allele carriers showed no (a; sample 1) CS differentiation or even inverted differentiation favoring the CS− (b; sample 2). During late acquisition (right), this pattern reversed with enhanced responding towards the CS+ compared to the CS- in the Risk group only. Risk group status was defined as carrying at least one risk allele (A allele). CS+: stimulus that was followed by the unconditioned stimulus; CS-: stimulus that was never followed by the unconditioned stimulus. R, right; L, left. Threshold at *P*=0.005 for illustrative purposes.

*Sample 1*. Discrimination between CS+ and CS− was on a neural level attenuated for the Risk group compared to the No-Risk group in the left amygdala, hippocampus, and insula during the full course of fear acquisition. This attenuation was further observed in the right anterior cingulate, medial orbitofrontal and superior frontal gyrus and insula during early acquisition. During late acquisition, however, stronger CS+>CS- discrimination for the Risk group was observed in the right thalamus (Table 2, Figure 2), while for the No-Risk group stronger discrimination was observable in the hippocampus. Partly overlapping findings were found for the Combined Risk group definition (Supplementary Table S9).

*Sample 2*. Analyses yielded an interaction between group and CS that was specific for fear acquisition, while no effects were found for the extinction. During early acquisition and in line with data from sample 1, subjects with at least one risk allele showed enhanced defensive responding encompassing neural activation in areas such as the left insula, bilateral ACC, amygdala, thalamus, and right midbrain towards presentation of the CS- compared to the CS+, while the No-Risk group showed a normal pattern of conditioning (stronger responses towards the CS+ than the CS-). During late acquisition, this pattern reversed and showed neural indicators of sustained differential conditioning in the Risk group towards the CS+ while the No-Risk group showed decreased differential CS responding (Table 2, Figure 2). No differential activation was found during the extinction phase. Similar findings were obtained for the Combined Risk group definition (Supplementary Table S11).

### *GLRB* modulation of brain morphology

**Figure 3:**
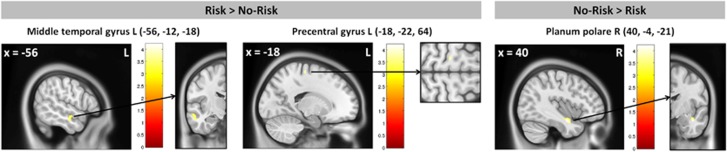
Volumetric group effect for Risk vs No-Risk group from sample 1 as assessed by exploratory voxel-based morphometry. Display threshold at *P*=0.001, k<= 15. Risk group status was defined as carrying at least one risk allele (A allele). R, right; L, left.

*Sample 1*. Genotype-dependent structural alterations were observed in regions of interest as increased grey matter volume for the Risk group in the right superior medial frontal gyrus and left precentral gyrus. Results of the exploratory whole brain analysis revealed increased grey matter volume for the Risk group in the precentral and superior medial frontal gyrus and middle frontal gyrus, middle temporal gyrus, superior temporal gyrus and angular gyrus while reductions were observed in the planum polare (Figure 3, Supplementary Table S4). Findings encompassing the right superior medial frontal gyrus, left precentral gyrus and left middle frontal gyrus (Risk>No-Risk), as well as right planum temporale (No-Risk>Risk) were replicated in the combined risk group analysis (Supplementary Table S9).

### *GLRB* modulation of startle reflex reactivity

*Sample 1*. The interaction between time x genotype-group did not reveal differential decrements in responding between both genotype groups (F (1,103)=0.526, *P*=0.470, see Supplementary Table S5 for statistical details). The startle effect could not be replicated using the combined risk group definition (Supplementary Table S12).

## Discussion

Recent findings^1^ reported strong translational evidence for glycine neurotransmission as a modulator of the brain’s evolutionary old defensive system and provided a strong, biologically plausible candidate intermediate phenotype of defensive reactivity within the domain of negative valence and arousal systems as laid out in the RDoC matrix.

The present paper conceptually replicated and extended these initial findings on the behavioral, neurofunctional and neurostructural level, yielding the following major findings: converging evidence was found for increased anterior insular activation in *GLRB* risk allele carriers (rs7688285) in both samples despite two procedurally different fear conditioning tasks, thus supporting the initial report.^1^ Also in line, we showed increased differential conditioning (CS+>CS−) during late acquisition in the *GLRB* risk groups on a neurofunctional level. Extending previous evidence, *GLRB* risk allele carriers showed an inverse pattern of responding by increased processing of the CS− compared with the CS+ during the early acquisition. These findings were also observed when using an alternative Combined Risk group definition previously employed^1^ (see Supplementary Information), thus further supporting their robustness. Results on startle reactivity were inconclusive and demand further investigations.

### General reactivity of the brain’s defensive system as a function of the *GLRB* genotype

The insular cortex serves as a cortical representation of upstream visceral information.^38^ Meta-analytic evidence assigns a central role of the anterior insula to fear conditioning in healthy subjects^11, 12^ and implicates the insula in pathological states of anxiety.^39^ We here provide converging evidence that allelic variation in the *GLRB* gene is associated with stronger activation of the anterior insula during fear conditioning, being in line with the previous report.^1^ Beyond this insular focus, some other areas of interest from this report, including the precentral gyrus, amygdala, and basal ganglia, could be replicated in sample 2, while replication failed for other brain regions such as the thalamus or hippocampus, possibly due to procedural differences in study designs. Noteworthy, stronger pons activation in *GLRB* risk carriers was observed in sample 2 during extinction. Volume enlargement of the superior medial frontal and precentral gyrus was detected in the structural dataset, thus providing preliminary evidence for *GLRB* modulation of brain structure. Despite an overlap of morphometric differences between genotype groups in these areas, functional activation differences observed in the same ROIs are unlikely to be attributed to structural differences, as they rely on differential within-subject contrasts (CS+>CS−).

Glycine receptors play a major role for inhibitory neurotransmission. Single-point mutations in genes encoding glycine receptors, including its beta receptor subunit (*GLRB*), have been associated with human hyperekplexia, or startle disease.^40, 41^ This condition is characterized by exaggerated startle reflexes in response to sudden unexpected auditory, visual or tactile stimuli. On a translational level, this phenotype is confirmed by the spastic mouse (featuring a substantial reduction of *Glrb*), showing exaggerated startle reactions^42, 43^ as a basic indicator of defensive system reactivity. Evidence on attenuated startle habituation in *GLRB* risk allele carriers in the present analysis was however negative. Paralleling previous findings^1^ on a descriptive level, we observed different slopes of startle habituation according to *GLRB* allelic variation. Single-trial data (Supplementary Figure S4) suggest higher initial startle reactivity in risk-allele carriers that might manifest as attenuated habituation in blocked analyses. Future studies are needed to elucidate the underlying processes of *GLRB* dependent startle reactivity in depth.

Being more ancient than the GABA system, glycine receptors are mainly distributed in evolutionary older brain regions encompassing the spinal cord, brainstem including the midbrain, and olfactory bulb,^44, 45^ but recent transcript analyses detected GlyB receptors also in the frontal cortex, hippocampus and striatum.^46^ As shown in the previous report^1^ genotype-specific differences of the promoter region risk variant rs7688285 on mRNA expression levels were found in the midbrain, where the minor, risk (A)-allele increased the mean expression of *GLRB* significantly by 1.34 fold. Neither in the forebrain nor in the amygdalae, rs7688285 affected mRNA expression. Transcript and protein determination of *Glrb* in spastic mice pointed to low transcript expression of *Glrb* particularly in the cortex and thalamus.^1^ We assume that our findings reflect a bottom-up modulatory effect on higher-order defensive system components by more hard-wired reflexes in the brainstem (as reflected by pons and midbrain activation in sample 2), which is corroborated by high-resolution diffusion imaging^47^ and resting-state^48^ fMRI studies showing that the insula is functionally connected to the brainstem and that coma-causing brain stem lesions are associated with a dissociation of this network.^48^

Altogether with the previous report, we provide converging evidence from a total of three different samples on the effect of allelic variation in the *GLRB* gene for enhanced activation of the anterior insula as a core component of higher-order threat-processing systems. However, the *GLRB* Risk group did not report higher symptoms of anxiety or showed different subjective or autonomic conditionability. This observation is in line with present findings representing an intermediate phenotype, where variance in defensive system responding is related to allelic variation of the *GLRB*, yet does not lead to symptoms of pathological anxiety. We thus provide further support for the role of *GLRB*, which is listed under the arousal gene list in the RDoC matrix, for a neurofunctional intermediate phenotype associated with enhanced defensive system reactivity that may increase the vulnerability to develop pathological forms of anxiety.

### Differential reactivity of the brain’s defensive system as a function of the *GLRB* genotype

Fear conditioning is a basic learning mechanism that allows the organism to flexibly adapt to environmental dangers. Importantly, during differential fear conditioning, the organism learns to process the CS+ as a threat signal, while associating the unreinforced CS- with safety.^49^ Depending on the stimulus presented, subjects will either activate the defensive system towards the CS+, or inhibit it towards the CS-. Despite procedural differences across the fear conditioning tasks, we observed surprisingly strong evidence for enhanced CS discrimination (CS+>CS-) in *GLRB* risk allele carriers from both samples particularly during the late phase of fear conditioning in fear processing regions such as the thalamus (sample 1) or amygdala, hippocampus, ACC, and insula (sample 2), showing an overlap particularly in the insula and thalamus with previously reported findings.^1^ Extending these, we additionally observed an inverse effect during early fear conditioning: here the *GLRB* risk group showed impaired CS- discrimination (sample 1: precentral gyrus and ACC) or even enhanced responding towards the CS− (sample 2: ACC, insula, amygdala, thalamus, and midbrain) that resembles impaired fear inhibitory learning as reported for pathological states of anxiety. While the main group effect of *GLRB* was driven by enhanced responding in the insula during both acquisition and extinction (sample 2), *GLRB* effects on differential conditioning were observed during fear learning only, thus pointing towards a specificity in modulating the acquisition of conditioned fear, but possibly not its recall or fear inhibitory learning (as induced during extinction training). Evidence from fear conditioning studies^50^ suggests that anxiety disorders (and particularly PD^51^) are not characterized by enhanced reactivity towards the CS+, but rather by attenuated fear inhibition in the face of a safety signal leading to reduced discrimination between both CSs. Using fMRI, enhanced activation of defensive circuit networks in response to a safety signal has been reported as a pathophysiological feature of PD^24, 52^ and as a marker of non-response towards exposure-based treatment^36^ that is based on fear-inhibitory learning, reflecting the relevance of safety signal processing for the etiopathogenesis of anxiety disorders. Given further evidence on the relevance of *GLRB* allelic variation regarding the underlying etiopathogenesis of anxiety disorders, it could represent an additional source in explaining altered patterns of fear conditioning, thus offering potential to stratify patient subgroups to individually tailored, novel therapeutic strategies (for example, fostering discriminatory learning).

### Study limitations

Owing to the post-hoc nature of this analysis, particularly sample 2 was possibly underpowered for subgroup analyses. However, in yielding a significant difference, these findings speak in favor of a large effect size of rs7688285 on brain activation which could be informative to base power calculations of future *a priori* studies upon. As not enough AA homozygote subjects were available in the fMRI samples, no dose–response relationship on the number of risk alleles could be investigated. fMRI and startle data were not available on the same sample, thus precluding multimodal comparisons across different units of analyses. Recent technical advances for combined assessments will allow for more fine-grained investigations of defensive system functioning in future studies.^53^ Data on brain morphology with sufficient resolution were only available for sample 1, but not sample 2 (due to restrictions in this multicenter study). Findings may serve as a starting point for further replication approaches on brain morphometric effects of *GLRB*. Although preliminary findings in the pons and midbrain were observed, it should be noted that the scanning parameters of both studies were not optimized for brain stem imaging, e.g. regarding pulsatory artifacts. Animal studies may furthermore inform about the interplay of these hierarchically organized systems and the modulatory function of the glycine receptors.

## Conclusion

Representing one of the first GWAS-confirmed findings related to the phenotype of PD^54, 55, 56^ and AG, converging evidence for *GLRB* allelic variation in defensive reactivity was derived from a large-scale translational approach, cross-cutting different levels of analyses. Of note, the robustness of this finding is demonstrated by consistent associations across different experimental paradigms and recording sites. As such, glycine-dependent neurotransmission may open up new avenues for mechanistic research on the etiopathogenesis of fear and anxiety disorders.

## Figures and Tables

**Table 1:** Main effect of *GLRB* Risk group on brain activation patterns during fear acquisition and extinction in sample 2 (cluster peak voxels are given)

*Contrast/region*	*Side*	*Voxels*	x	y	z	t	P^a^
*Overall: Risk>No-Risk*							
Insula	R	2387	34	14	14	4.49	<0.001
Insula^b^	L	33	−44	6	6	4.42	0.048
Precentral gyrus	L	554	−42	−8	46	4.26	<0.001
Superior parietal gyrus (2.83 mm dev.)	R	731	24	−52	48	3.91	<0.001
Precentral gyrus^b^	R	164	48	−2	46	3.62	0.040
Middle occipital gyrus (2.00 mm dev.)	R	204	40	−68	0	3.54	<0.001
Inferior frontal operculum	L	276	−44	8	6	3.42	<0.001
Superior frontal gyrus	R	179	28	−8	64	2.98	0.002
*Overall: No-Risk>Risk*	No differential activation						
*Full acquisition: Risk>No-Risk*							
Middle occipital gyrus	R	375	40	−68	2	4.36	<0.001
Precentral gyrus	L	284	−42	−8	46	3.97	<0.001
Rolandic operculum (4.00 mm dev.)	R	805	42	−16	26	3.84	<0.001
Precentral gyrus	R	354	38	−22	70	3.59	<0.001
Insula	R	660	32	14	14	3.41	<0.001
*Full acquisition: No-Risk>Risk*	No differential activation						
*Full extinction: Risk>No-Risk*							
Precuneus (2.00 mm dev.)	R	993	20	−40	46	4.41	<0.001
Insula (2.00 mm dev.)	R	801	32	26	14	4.27	<0.001
Caudate nucleus (2.00 mm dev.)	R	144	16	−10	26	3.81	<0.001
Pons^b^	R	28	4	−22	−32	3.66	0.017
Insula	L	385	−40	6	−4	3.50	<0.001
Amygdala^b^	L	2	−22	2	−24	3.49	0.006
Precentral gyrus	L	225	−40	−10	52	3.09	0.001
*Full extinction: No-Risk>Risk*	No differential activation						

Abbreviations: dev, Deviation (in mm) from the identified anatomical structure using anatomic automatic labeling (aal); L, left; R, right; voxel, number of voxels per cluster; *x*, *y*, *z*, MNI coordinates.

a Whole brain results at *P*<0.005 (uncorr.) with a minimum cluster size of 142 contiguous voxels, indicating to correct for multiple comparisons at *P*<0.05.

b Small volume correction using aal masks (family-wise error correction at *P*<0.05) with a cluster forming threshold of *P*<0.001.

Risk group status was defined as carrying at least one risk allele (A allele).

**Table 2:** Interaction effect *GLRB* Risk group and conditioned stimulus (CS+ vs CS−) on brain activation patterns during fear acquisition

*Contrast/region*	*Side*	*Voxels*	x	y	z	t	P
**Sample 1**							
*Full acquisition: Risk>No-Risk (CS+>CS−)*						No differential activation	
*Full acquisition: Risk>No-risk (CS->CS+)*							
Amygdala	L	13	−30	−4	−18	4.39	0.002
Hippocampus	L	13	−26	−14	−24	3.93	0.016
Hippocampus	L	13	−28	−8	−22	3.54	0.043
*Early acquisition: Risk>No-Risk (CS+>CS−)*						No differential activation	
*Early acquisition: Risk>No-Risk (CS−>CS+)*							
Precentral gyrus	L	38	−58	−2	22	4.21	0.018
Anterior cingulate gyrus	R	4	4	34	2	3.95	0.025
Middle cingulate gyrus	R	7	2	−6	30	3.80	0.036
*Late acquisition: Risk>No-Risk* (CS+>CS−)							
Thalamus	R	14	18	−12	8	4.33	0.009
*Late acquisition: Risk>No-Risk* (CS->CS+)						No differential activation	
**Sample 2**							
*Full acquisition: Risk>No-Risk (CS+>CS−)*						No differential activation	
*Full acquisition: Risk>No-Risk (CS−>CS+)*							
Thalamus^a^	L	28	0	−20	6	4.08	0.003
Thalamus^a^	R	10	2	−20	6	3.81	0.007
*Early acquisition: Risk>No-Risk (CS+>CS−)*						No differential activation	
*Early acquisition: Risk>No-Risk* (CS−>CS+)							
Middle temporal gyrus	R	271	58	0	−20	5.37	<0.001
Postcentral gyrus	R	4982	28	−48	−72	4.68	<0.001
Middle occipital gyrus	L	724	−34	−85	36	4.58	<0.001
Thalamus	L	364	0	−20	5	4.57	<0.001
Thalamus^a^	R	48	2	−20	6	4.43	0.001
Amygdala	L	4114	−24	−4	−16	4.28	<0.001
Superior temporal gyrus	L	785	−58	−5	0	3.90	<0.001
Hippocampus^a^	L	114	−24	−8	−18	3.96	0.005
Postcentral gyrus	R	453	66	−18	38	3.78	<0.001
Anterior cingulate gyrus^a^	L	84	0	42	4	3.69	0.016
Anterior cingulate gyrus^a^	R	55	2	42	4	3.73	0.012
Anterior cingulate gyrus^a^	R	12	8	8	28	3.68	0.014
Supplementary motor area^a^	L	22	2	8	44	3.65	0.022
Supplementary motor area	R	189	8	−16	76	3.62	<0.001
Anterior cingulate gyrus^a^	L	3	2	18	−10	3.50	0.030
Anterior cingulate gyrus^a^	R	41	4	28	28	3.52	0.024
Insula^a^	L	14	−26	10	−20	3.52	0.035
Superior medial frontal gyrus^a^	R	10	2	46	2	3.51	0.038
Anterior cingulate gyrus^a^	L	49	0	22	26	3.47	0.033
Middle frontal gyrus	R	211	30	50	18	3.45	<0.001
Medial orbitofrontal gyrus^a^	R	1	0	22	−12	3.44	0.020
Midbrain^a^	R	15	12	−28	−12	3.43	0.046
Amygdala^a^	R	10	24	4	−20	3.28	0.014
Medial orbitofrontal gyrus^a^	L	2	−4	22	−14	3.20	0.035
*Late acquisition: Risk>No-Risk*(*CS+>CS*−)							
Temporal pole	R	445	40	8	−38	4.31	<0.001
Middle temporal gyrus	R	503	52	−68	2	4.24	<0.001
Vermis 4 5	–	2983	6	−54	2	4.09	<0.001
Rolandic operculum	R	1251	50	−14	18	3.92	<0.001
Inferior frontal operculum	R	95760	60	14	14	3.88	<0.001
Pons^a^	R	42	10	−32	−26	3.87	0.010
Anterior cingulate gyrus	L	735	−2	22	26	3.86	<0.001
Middle frontal gyrus	R	178	26	40	28	3.85	<0.001
Superior frontal gyrus	R	352	34	−2	68	3.83	<0.001
Anterior cingulate gyrus^a^	R	29	2	20	28	3.65	0.016
Middle temporal gyrus (2.00 mm dev.)	R	438	48	−16	−16	3.62	<0.001
Cuneus	R	753	14	86	28	3.58	<0.001
Amygdala^a^	R	8	32	2	−30	3.47	0.008
Hippocampus (2.83 mm dev.)	L	161	−38	10	−18	3.38	<0.001
Hippocampus^a^	R	4	42	−16	−14	3.30	0.041
Insula (2.00 mm dev.)	R	278	28	20	−8	3.26	0.001
Middle frontal gyrus	L	149	−26	14	46	3.17	0.001
*Late acquisition: Risk>No-risk (CS−>CS+)*						No differential activation	

Abbreviations: CS: conditioned stimulus; CS-: CS that is never followed by an unconditioned stimulus (US); CS+: CS that is always followed by the US (sample 1) or with a reinforcement rate of 50% (sample 2; only unpaired CS+ were included in the analysis); L: left; R: right; voxel: number of voxels per cluster; x, y, z: MNI coordinates; dev.: deviation (in mm) from the identified anatomical structure using anatomic automatic labeling (aal).

Risk group status was defined as carrying at least one risk allele (A allele).

Sample 1: ROI peak voxels are given. Small volume correction in pre-defined ROI analyses (family-wise error correction at *P*<0.05) with a cluster forming threshold of *P*<0.001.

Sample 2: Whole-brain results at *P*<0.005 (uncorr.) with a minimum cluster size of 142 contiguous voxels, indicating to correct for multiple comparisons at *P*<0.05.

a Small volume correction using aal masks (family-wise error correction at *P*<0.05) with a cluster forming threshold of *P*<0.001. No significant clusters were detected for the extinction phase (full, early, or late).

## References

[bib1] Deckert J, Weber H, Villmann C, Lonsdorf TB, Richter J, Andreatta M et al. *GLRB*allelic variation predisposes to agoraphobia by increasing startle response. Mol Psychiatry 2017; doi: 10.1038/mp.2017.2 (e-pub ahead of print). 10.1038/mp.2017.2 28167838

[bib2] Insel TR, Cuthbert BN, Garvey M, Heinssen R, Pine DS, Quinn K et al. Research domain criteria (RDoC): toward a new classification framework for research on mental disorders. Am J Psychiatry 2010; 167: 748–751. 20595427 10.1176/appi.ajp.2010.09091379

[bib3] Stroebe W, Strack F. The Alleged crisis and the illusion of exact replication. Perspect Psychol Sci 2014; 9: 59–71. 26173241 10.1177/1745691613514450

[bib4] Open Science Collaboration. Estimating the reproducibility of psychological science. Science 2015; 349: aac4716. 26315443 10.1126/science.aac4716

[bib5] Fanselow MS. Neural organization of the defensive behavior system responsible for fear. Psychonom Bull Rev 1994; 1: 429–438. 10.3758/BF03210947 24203551

[bib6] Blanchard DC, Hynd AL, Minke KA, Minemoto T, Blanchard RJ. Human defensive behaviors to threat scenarios show parallels to fear- and anxiety-related defense patterns of non-human mammals. Neurosci Biobehav Rev 2001; 25: 761–770. 11801300 10.1016/s0149-7634(01)00056-2

[bib7] Mobbs D, Hagan CC, Dalgleish T, Silston B, Prevost C. The ecology of human fear: survival optimization and the nervous system. Front Neurosci 2015; 9: 55. 25852451 10.3389/fnins.2015.00055 PMC4364301

[bib8] McNaughton N, Corr PJ. A two-dimensional neuropsychology of defense: fear/anxiety and defensive distance. Neurosci Biobehav Rev 2004; 28: 285–305. 15225972 10.1016/j.neubiorev.2004.03.005

[bib9] Mobbs D, Petrovic P, Marchant JL, Hassabis D, Weiskopf N, Seymour B et al. When fear is near: threat imminence elicits prefrontal-periaqueductal gray shifts in humans. Science 2007; 317: 1079–1083. 17717184 10.1126/science.1144298 PMC2648508

[bib10] Mobbs D, Marchant JL, Hassabis D, Seymour B, Tan G, Gray M et al. From threat to fear: The neural organization of defensive fear systems in humans. J Neurosci 2009; 29: 12236–12243. 19793982 10.1523/JNEUROSCI.2378-09.2009 PMC2782300

[bib11] Sehlmeyer C, Schoning S, Zwitserlood P, Pfleiderer B, Kircher T, Arolt V et al. Human fear conditioning and extinction in neuroimaging: A systematic review. PLoS ONE 2009; 4: e5865. 19517024 10.1371/journal.pone.0005865 PMC2692002

[bib12] Fullana MA, Harrison BJ, Soriano-Mas C, Vervliet B, Cardoner N, Avila-Parcet A et al. Neural signatures of human fear conditioning: an updated and extended meta-analysis of fMRI studies. Mol Psychiatry 2016; 21: 500–508. 26122585 10.1038/mp.2015.88

[bib13] Fredrikson M, Annas P, Hettema JM. Different genetic factors underlie fear conditioning and episodic memory. Psychiatr Genet 2015; 25: 155–162. 25967536 10.1097/YPG.0000000000000088

[bib14] Hettema JM, Annas P, Neale MC, Kendler KS, Fredrikson M. A twin study of the genetics of fear conditioning. Arch Gen Psychiatry 2003; 60: 702–708. 12860774 10.1001/archpsyc.60.7.702

[bib15] Royce JR. Avoidance conditioning in nine strains of inbred mice using optimal stimulus parameters. Behav Genet 1972; 2: 107–110. 4664197 10.1007/BF01066739

[bib16] Gordon JA, Hen R. Genetic approaches to the study of anxiety. Annu Rev Neurosci 2004; 27: 193–222. 15217331 10.1146/annurev.neuro.27.070203.144212

[bib17] Anokhin AP, Golosheykin S, Heath AC. Genetic and environmental influences on emotion-modulated startle reflex: a twin study. Psychophysiology 2007; 44: 106–112. 17241146 10.1111/j.1469-8986.2006.00486.x

[bib18] Hasenkamp W, Epstein MP, Green A, Wilcox L, Boshoven W, Lewison B et al. Heritability of acoustic startle magnitude, prepulse inhibition, and startle latency in schizophrenia and control families. Psychiatry Res 2010; 178: 236–243. 20483176 10.1016/j.psychres.2009.11.012 PMC2902662

[bib19] Blokland GA, de Zubicaray GI, McMahon KL, Wright MJ. Genetic and environmental influences on neuroimaging phenotypes: a meta-analytical perspective on twin imaging studies. Twin Res Hum Genet 2012; 15: 351–371. 22856370 10.1017/thg.2012.11 PMC4291185

[bib20] Koten JW Jr, Wood G, Hagoort P, Goebel R, Propping P, Willmes K et al. Genetic contribution to variation in cognitive function: an FMRI study in twins. Science 2009; 323: 1737–1740. 19325117 10.1126/science.1167371

[bib21] Manuck SB, Brown SM, Forbes EE, Hariri AR. Temporal stability of individual differences in amygdala reactivity. Am J Psychiatry 2007; 164: 1613–1614. 17898358 10.1176/appi.ajp.2007.07040609

[bib22] Sheehan DV, Lecrubier Y, Sheehan KH, Amorim P, Janavs J, Weiller E et al. The Mini-International Neuropsychiatric Interview (M.I.N.I.): the development and validation of a structured diagnostic psychiatric interview for DSM-IV and ICD-10. J Clin Psychiatry 1998; 59(Suppl 20): 22–33. 9881538

[bib23] Gloster AT, Wittchen HU, Einsle F, Hofler M, Lang T, Helbig-Lang S et al. Mechanism of action in CBT (MAC): methods of a multi-center randomized controlled trial in 369 patients with panic disorder and agoraphobia. Eur Arch Psychiatry Clin Neurosci 2009; 259(Suppl 2): S155–S166. 19876674 10.1007/s00406-009-0065-6

[bib24] Lueken U, Straube B, Reinhardt I, Maslowski NI, Wittchen HU, Strohle A et al. Altered top-down and bottom-up processing of fear conditioning in panic disorder with agoraphobia. Psychol Med 2014; 44: 381–394. 23611156 10.1017/S0033291713000792

[bib25] Wittchen HU, Pfister H. DIA-X Interview. Swets & Zeitlinger: Frankfurt, 1997.

[bib26] Haaker J, Lonsdorf TB, Thanellou A, Kalisch R. Multimodal assessment of long-term memory recall and reinstatement in a combined cue and context fear conditioning and extinction paradigm in humans. PLoS ONE 2013; 8: e76179. 24116095 10.1371/journal.pone.0076179 PMC3792118

[bib27] Kuhn M, Haaker J, Glotzbach-Schoon E, Schumann D, Andreatta M, Mechias ML et al. Converging evidence for an impact of a functional NOS gene variation on anxiety-related processes. Soc Cogn Affect Neurosci 2016; 11: 803–812. 26746182 10.1093/scan/nsv151 PMC4847690

[bib28] Lonsdorf TB, Haaker J, Kalisch R. Long-term expression of human contextual fear and extinction memories involves amygdala, hippocampus and ventromedial prefrontal cortex: a reinstatement study in two independent samples. Soc Cogn Affect Neurosci 2014; 9: 1973–1983. 24493848 10.1093/scan/nsu018 PMC4249485

[bib29] Ashburner J. A fast diffeomorphic image registration algorithm. Neuroimage 2007; 38: 95–113. 17761438 10.1016/j.neuroimage.2007.07.007

[bib30] Tzourio-Mazoyer N, Landeau B, Papathanassiou D, Crivello F, Etard O, Delcroix N et al. Automated anatomical labeling of activations in SPM using a macroscopic anatomical parcellation of the MNI MRI single-subject brain. Neuroimage 2002; 15: 273–289. 11771995 10.1006/nimg.2001.0978

[bib31] Maldjian JA, Laurienti PJ, Kraft RA, Burdette JH. An automated method for neuroanatomic and cytoarchitectonic atlas-based interrogation of fMRI data sets. Neuroimage 2003; 19: 1233–1239. 12880848 10.1016/s1053-8119(03)00169-1

[bib32] Kircher T, Arolt V, Jansen A, Pyka M, Reinhardt I, Kellermann T et al. Effect of cognitive behavioural therapy on neural correlates of fear conditioning in panic disorder. Biol Psychiatry 2013; 73: 93–101. 22921454 10.1016/j.biopsych.2012.07.026

[bib33] Bradley MM, Lang PJ. Measuring emotion - the self-assessment mannequin and the semantic differential. J Behav Ther Exp Psychiatr 1994; 25: 49–59. 10.1016/0005-7916(94)90063-9 7962581

[bib34] Beck AT, Steer RA, Brown GK. Beck Depression Inventory, 2nd ed. The Psychological Corporation: San Antonio, 1996.

[bib35] Friedman L, Glover GH, Krenz D, Magnotta V. Reducing inter-scanner variability of activation in a multicenter fMRI study: role of smoothness equalization. Neuroimage 2006; 32: 1656–1668. 16875843 10.1016/j.neuroimage.2006.03.062

[bib36] Lueken U, Straube B, Konrad C, Wittchen HU, Strohle A, Wittmann A et al. Neural substrates of treatment response to cognitive-behavioral therapy in panic disorder with agoraphobia. Am J Psychiatry 2013; 170: 1345–1355. 23982225 10.1176/appi.ajp.2013.12111484

[bib37] Slotnick SD, Moo LR, Segal JB, Hart J Jr.. Distinct prefrontal cortex activity associated with item memory and source memory for visual shapes. Brain Res Cogn Brain Res 2003; 17: 75–82. 12763194 10.1016/s0926-6410(03)00082-x

[bib38] Craig AD. How do you feel–now? The anterior insula and human awareness. Nat Rev Neurosci 2009; 10: 59–70. 19096369 10.1038/nrn2555

[bib39] Etkin A, Wager TD. Functional neuroimaging of anxiety: a meta-analysis of emotional processing in PTSD, social anxiety disorder, and specific phobia. Am J Psychiatry 2007; 164: 1476–1488. 17898336 10.1176/appi.ajp.2007.07030504 PMC3318959

[bib40] Chung SK, Bode A, Cushion TD, Thomas RH, Hunt C, Wood SE et al. *GLRB* is the third major gene of effect in hyperekplexia. Hum Mol Gen 2013; 22: 927–940. 23184146 10.1093/hmg/dds498

[bib41] James VM, Bode A, Chung SK, Gill JL, Nielsen M, Cowan FM et al. Novel missense mutations in the glycine receptor beta subunit gene (*GLRB* in startle disease. Neurobiol Dis 2013; 52: 137–149. 23238346 10.1016/j.nbd.2012.12.001 PMC3581774

[bib42] Becker CM. Disorders of the inhibitory glycine receptor: the spastic mouse. FASEB J 1990; 4: 2767–2774. 2165011 10.1096/fasebj.4.10.2165011

[bib43] Schaefer N, Langlhofer G, Kluck CJ, Villmann C. Glycine receptor mouse mutants: model systems for human hyperekplexia. British J Pharmacol 2013; 170: 933–952. 10.1111/bph.12335 PMC3949644 23941355

[bib44] Lynch JW. Molecular structure and function of the glycine receptor chloride channel. Physiol Rev 2004; 84: 1051–1095. 15383648 10.1152/physrev.00042.2003

[bib45] Weltzien F, Puller C, O'Sullivan GA, Paarmann I, Betz H. Distribution of the glycine receptor beta-subunit in the mouse CNS as revealed by a novel monoclonal antibody. J Comp Neurol 2012; 520: 3962–3981. 22592841 10.1002/cne.23139

[bib46] Jonsson S, Morud J, Pickering C, Adermark L, Ericson M, Soderpalm B. Changes in glycine receptor subunit expression in forebrain regions of the Wistar rat over development. Brain Res 2012; 1446: 12–21. 22330726 10.1016/j.brainres.2012.01.050

[bib47] Park S, Tyszka JM, Allman JM. The claustrum and insula in Microcebus murinus: a high resolution diffusion imaging study. Front Neuroanatomy 2012; 6: 21. 10.3389/fnana.2012.00021 PMC3374366 22707933

[bib48] Fischer DB, Boes AD, Demertzi A, Evrard HC, Laureys S, Edlow BL et al. A human brain network derived from coma-causing brainstem lesions. Neurology 2016; 87: 2427–2434. 27815400 10.1212/WNL.0000000000003404 PMC5177681

[bib49] Christianson JP, Fernando ABP, Kazama AM, Jovanovic T, Ostroff LE, Sangha S. Inhibition of fear by learned safety signals: minisymposium review. J Neurosci 2012; 32: 14118–14124. 23055481 10.1523/JNEUROSCI.3340-12.2012 PMC3541026

[bib50] Duits P, Cath DC, Lissek S, Hox JJ, Hamm AO, Engelhard IM et al. Updated meta-analysis of classical fear conditioning in the anxiety disorders. Depress Anxiety 2015; 32: 239–253. 25703487 10.1002/da.22353

[bib51] Lissek S, Rabin SJ, McDowell DJ, Dvir S, Bradford DE, Geraci M et al. Impaired discriminative fear-conditioning resulting from elevated fear responding to learned safety cues among individuals with panic disorder. Behav Res Ther 2009; 47: 111–118. 19027893 10.1016/j.brat.2008.10.017 PMC2758527

[bib52] Tuescher O, Protopopescu X, Pan H, Cloitre M, Butler T, Goldstein M et al. Differential activity of subgenual cingulate and brainstem in panic disorder and PTSD. J Anxiety Disord 2011; 25: 251–257. 21075593 10.1016/j.janxdis.2010.09.010 PMC4096628

[bib53] Lindner K, Neubert J, Pfannmoller J, Lotze M, Hamm AO, Wendt J. Fear-potentiated startle processing in humans: Parallel fMRI and orbicularis EMG assessment during cue conditioning and extinction. Int J Psychophysiol 2015; 98(3 Pt 2): 535–545. 25725377 10.1016/j.ijpsycho.2015.02.025

[bib54] Quast C, Altmann A, Weber P, Arloth J, Bader D, Heck A et al. Rare variants in TMEM132D in a case-control sample for panic disorder. Am J Med Genet 2012; 159b: 896–907. 22911938 10.1002/ajmg.b.32096

[bib55] Erhardt A, Czibere L, Roeske D, Lucae S, Unschuld PG, Ripke S et al. TMEM132D, a new candidate for anxiety phenotypes: evidence from human and mouse studies. Mol Psychiatry 2011; 16: 647–663. 20368705 10.1038/mp.2010.41

[bib56] Haaker J, Lonsdorf TB, Raczka KA, Mechias ML, Gartmann N, Kalisch R. Higher anxiety and larger amygdala volumes in carriers of a TMEM132D risk variant for panic disorder. Transl Psychiatry 2014; 4: e357. 24495968 10.1038/tp.2014.1 PMC3944634

