# Supplementary material for: Modulation of defensive reactivity by GLRB allelic variation: converging evidence from an intermediate phenotype approach
Source: Transl Psychiatry. 2017 Sep 5;7(9):e1227–. doi: 10.1038/tp.2017.186 (PMC5639239; doi:10.1038/tp.2017.186)
Supplement: Supplementary Information [file tp2017186x1.docx]

**Supplementary Information**

For the manuscript: “Modulation of defensive reactivity by *GLRB* allelic variation: Converging evidence from an intermediate phenotype approach”

**Supplement contents:**

Supplementary information for the *GLRB* Risk groups as classified for the rs7688285 single nucleotide polymorphism (SNP) as used in the main article:

- Sample characteristics of samples 1 and 2 (Table S1)
- Supplementary results on *GLRB* modulation of fear conditioning: statistical details and visualization for behavioral measures of fear conditioning of sample 1 (Table S2; Figure S1)
- Supplementary results on *GLRB* modulation of fear conditioning: Statistical details and visualization for behavioral measures of fear conditioning of sample 2 (Table S3; Figure S2)
- Supplementary results on *GLRB* modulation of brain morphology (Table S4)
- Supplementary results on *GLRB* modulation of startle reflex reactivity: full statistical information (Table S5, Figure S3) and single trial inter-trial interval (ITI) response (Figure S4).

Supplementary results for the *GLRB* Combined Risk groups (combined risk: at least one risk allele in rs17035763, rs191260602, rs7688285, or rs78726293):

- Sample characteristics for the Combined Risk group from samples 1 and 2 (Table S6)
- Functional and structural MRI results on *GLRB* modulation for the Combined Risk group from sample 1 (Tables S7 - S9)
- Functional MRI results on *GLRB* modulation for the Combined Risk group from sample 2 (Tables S10, S11)
- Startle reflex reactivity on *GLRB* modulation for the Combined Risk group from sample 1 (Table S12)

**Supplementary figure legends**

**Supplementary Figure S1.** (A) Subjective fear ratings of fear/stress/tension and (B) skin conductance reactions (SCRs) for sample 1. Risk group status was defined as carrying at least one risk allele (A allele).

**Supplementary Figure S2.** Valence ratings (upper half) and arousal ratings (lower half) of the fear conditioning paradigm of sample 2. Risk group status was defined as carrying at least one risk allele (A allele).

**Supplementary Figure S3.** Inter-trial interval (ITI) startle blink magnitudes (T-Scores) divided by the first and second time block (blocl1, block2) of the experiment for Risk vs. No-Risk group in sample 1. Risk group status was defined as carrying at least one risk allele (A allele).

**Supplementary Figure S4.** Single trial inter-trial interval (ITI) responses per Risk group in sample 1. Risk group status was defined as carrying at least one risk allele (A allele).
