# Supplementary material for: Modulation of defensive reactivity by GLRB allelic variation: converging evidence from an intermediate phenotype approach
Source: Transl Psychiatry. 2017 Sep 5;7(9):e1227–. doi: 10.1038/tp.2017.186 (PMC5639239; doi:10.1038/tp.2017.186)
Supplement: Supplementary Table 1 [file tp2017186x2.docx]

| **Table S1.** Demographic and clinical characteristics of samples 1 and 2. Means (SD) except where noted. | | | | | | | |
| --- | --- | --- | --- | --- | --- | --- | --- |
|  | **sample 1** | | | | | Chi^2^ or t (df) | p |
| fMRI fear conditioning sample (n = 48)^1^ | Risk allele carriers  (AA, AG; n = 18 [37.5%]) | | No-Risk allele carriers  (GG; n = 30 [62.5%]) | | |  |  |
| Female gender [n (%)] | 10 | (55.56) | 14 | | (46.67) | 0.089 (1) | 0.766 |
| Years of education [n (%)] |  |  |  | |  |  |  |
| 8 | 0 | (0.00) | 0 | | (0.00) | n.a. | n.a. |
| 10 | 0 | (0.00) | 0 | | (0.00) |  |  |
| 12-13 | 18 | (100.00) | 30 | | (100.00) |  |  |
| NOS1ex1f s-carrier [n (%)] | 11 | (61.11) | 14 | | (46.67) | 0.451 (1) | 0.502 |
| Age (years) | 23.33 | (2.06) | 25.90 | | (3.40) | 2.895 (46) | 0.006 |
| US intensity (mA) | 6.03 | (4.03) | 5.86 | | (3.65) | -0.152 (46) | 0.880 |
| ASI total | 13.78 | (5.68) | 14.60 | | (7.15) | 0.415 (46) | 0.680 |
| Brain morphometry sample (n = 416)^2^ | Risk allele carriers  (AA, AG; n = 106 [25.5%]) | | No-Risk allele carriers  (GG; n = 310 [74.5%]) | | |  |  |
| Female gender [n (%)] | 31 | (29.24) | 84 | | (27.10) | 0.091 (1) | 0.763 |
| Years of education^3^ [n (%)] |  |  |  | |  |  |  |
| 8 | 0 | (0.00) | 2 | | (0.65) | 0.737 (2) | 0.692 |
| 10 | 4 | (3.77) | 13 | | (4.22) |  |  |
| 12-13 | 102 | (96.23) | 293 | | (95.13) |  |  |
| Age^4^ (years) | 25.28 | (5.09) | 26.04 | | (4.06) | 1.419 (411) | 0.157 |
| ASI total^5^ | 13.48 | (6.59) | 12.58 | | (6.27) | -1.265 (413) | 0.207 |
| Affective startle sample  (n = 105) ^5^ | Risk allele carriers  (AA, AG; n = 24 [22.9%]) | | No-Risk allele carriers  (GG; n = 81 [77.1%]) | | |  |  |
| Female gender^6^ [n (%)] | 13 | (56.52) | 44 | (54.32) | | < 0.001 (1) | 1.000 |
| Years of education^6^ [n (%)] |  |  |  |  | |  |  |
| 8 | 1 | (4.35) | 0 | (0.00) | | 3.813 (2) | 0.149 |
| 10 | 1 | (4.35) | 2 | (2.50) | |  |  |
| 12-13 | 21 | (91.30) | 79 | (97.53) | |  |  |
| Age^6^ (years) | 25.26 | (3.53) | 24.68 | (3.81) | | 0.656 (102) | 0.513 |
| ASI total^6^ | 14.87 | (7.33) | 15.00 | (7.340) | | 0.162 (101) | 0.941 |

|  | **sample 2** |  |  |
| --- | --- | --- | --- |

| fMRI fear conditioning sample (n = 38) | Risk allele carriers  (AA, AG; n = 17 [44.7%]) | | No-Risk allele carriers  (GG; n = 21 [55.3%]) | |  |  |
| --- | --- | --- | --- | --- | --- | --- |
| Female gender [n (%)] | 10 | (58.80) | 12 | (57.10) | 0.011 (1) | 0.917 |
| Years of education [n (%)] |  |  |  |  |  |  |
| 8 | 1 | (5.90) | 1 | (4.80) | 0.623 (2) | 0.732 |
| 10 | 5 | (29.40) | 4 | (19.00) |  |  |
| 12-13 | 11 | (64.70) | 16 | (76.20) |  |  |
| Site |  |  |  |  |  |  |
| Berlin | 6 | (35.30) | 9 | (42.90) | 7.633 (2) | 0.022 |
| Dresden | 3 | (17.60) | 10 | (47.60) |  |  |
| Münster | 8 | (47.10) | 2 | (9.50) |  |  |
| Age (years) | 34.88 | (9.44) | 38.00 | (11.88) | 0.880 (36) | 0.385 |
| Digit span forward | 7.88 | (2.06) | 8.29 | (2.00) | 0.610 (36) | 0.546 |
| Digit span backward | 6.82 | (2.16) | 6.57 | (2.34) | -0.342 (36) | 0.734 |
| TMT-A (sec) | 26.59 | (9.04) | 24.80 | (8.21) | -0.636 (36) | 0.529 |
| TMT-B (sec) | 58.41 | (17.32) | 57.24 | (19.27) | -0.195 (36) | 0.846 |
| US rating | 8.12 | (0.60) | 8.19 | (1.33) | 0.225 (29) | 0.824 |
| ASI total | 9.29 | (8.79) | 9.14 | (6.92) | -0.059 (36) | 0.953 |
| BDI II total | 0.88 | (1.22) | 2.05 | (2.29) | 2.007 (32) | 0.053 |

| ASI: Anxiety Sensitivity Index; BDI II: Beck Depression Inventory II; NOS1ex1f: *NOS1* ex1f-VNTR polymorphism; TMT-A: Trail Making Test A; TMT-B: Trail Making Test B; US intensity: unconditioned stimulus (in mA); US rating: US subjective ratings (10 point Likert Scale). n.a.: not applicable.  ^1^ The sample was initially recruited stratified for the *NOS1*-ex1f-VNTR genotype employing a combined cue and context fear conditioning paradigm during fMRI^1^. This sample (except for data of one subject due to missing genetic data) was re-analyzed with respect to the impact of *GLRB* genotype on neural activation to the cued acquisition of conditioned fear; ^2^ Structural data of subjects from the fMRI fear conditioning sample partly included; ^3^ n = 2 missing; ^4^ n = 3 missing; ^5^ original sample size n=107 (two data sets excluded due to missing genetic data); ^6^ n = 1 missing.  Note sample 1: genotype distribution for rs7688285 did not differ significantly from Hardy-Weinberg equilibrium in any of the samples (fear conditioning sample: p = 0.073; brain morphology sample: p = 0.227; affective startle sample: p = 0.072). All subjects of the fear condition and bran morphology samples and 7 subjects of the affective startle sample were included in the GWAS analyses of the previous report ^4^.  Note sample 2: genotype distribution did not differ significantly from the Hardy-Weinberg equilibrium (p = 0.158). |
| --- |
