# Supplementary material for: Modulation of defensive reactivity by GLRB allelic variation: converging evidence from an intermediate phenotype approach
Source: Transl Psychiatry. 2017 Sep 5;7(9):e1227–. doi: 10.1038/tp.2017.186 (PMC5639239; doi:10.1038/tp.2017.186)
Supplement: Supplementary Table 2 [file tp2017186x3.docx]

| **Table S2.** Statistical details for behavioral measures of sample 1 for subjective fear ratings of stress/fear/tension as well as SCRs as mean values of three acquisition blocks | | | |
| --- | --- | --- | --- |
|  | df | F | P |
| **Ratings** |  |  |  |
| CS-type | 1/46 | 73.996 | <0.001^1^ |
| *GLRB*-Risk | 1/46 | 0.164 | 0.688 |
| CS-type**GLRB*-Risk | 1/46 | 0.164 | 0.687 |
| **SCR** |  |  |  |
| CS-type | 1/39 | 10.522 | 0.002^1^ |
| *GLRB*-Risk | 1/39 | 1.280 | 0.265 |
| CS-type**GLRB*-Risk | 1/39 | 0.606 | 0.441 |
| Risk group status was defined as carrying at least one risk allele (A allele). CS: conditioned stimulus  ^1^ CS+ > CS- | | | |
