# Supplementary material for: Modulation of defensive reactivity by GLRB allelic variation: converging evidence from an intermediate phenotype approach
Source: Transl Psychiatry. 2017 Sep 5;7(9):e1227–. doi: 10.1038/tp.2017.186 (PMC5639239; doi:10.1038/tp.2017.186)
Supplement: Supplementary Table 3 [file tp2017186x4.docx]

| **Table S3.** Statistical details for behavioral measures of sample 2. Footnote denotes statistically significant contrasts based on post-hoc t-tests for pair-wise comparisons of significant effects. | | | |
| --- | --- | --- | --- |
|  | df | F | p |
| **Valence** |  |  |  |
| CS-type | 1/34 | 2.588 | 0.117 |
| Phase | 2/68 | 2.012 | 0.142 |
| *GLRB*-Risk | 1/34 | 0.176 | 0.677 |
| CS-type*Phase | 2/51 | 4.185 | 0.030 ^1^ |
| CS-type**GLRB*-Risk | 1/34 | 0.712 | 0.405 |
| Phase**GLRB*-Risk | 2/68 | 1.279 | 0.468 |
| CS-type*Phase**GLRB*-Risk | 2/51 | 2.131 | 0.141 |
| **Arousal** |  |  |  |
| CS-type | 1/34 | 3.301 | 0.078 |
| Phase | 2/68 | 0.912 | 0.407 |
| *GLRB*-Risk | 1/34 | 1.097 | 0.302 |
| CS-type*Phase | 2/51 | 3.449 | 0.056 |
| CS-type**GLRB*-Risk | 1/34 | 0.081 | 0.963 |
| Phase**GLRB*-Risk | 2/68 | 0.270 | 0.764 |
| CS-type*Phase**GLRB*-Risk | 2/51 | 0.082 | 0.855 |
| Risk group status was defined as carrying at least one risk allele (A allele). CS: conditioned stimulus.  ^1^ Acquisition: CS+ < CS-; CS+: Familiarization < Extinction, Acquisition < Extinction | | | |
