# Supplementary material for: Modulation of defensive reactivity by GLRB allelic variation: converging evidence from an intermediate phenotype approach
Source: Transl Psychiatry. 2017 Sep 5;7(9):e1227–. doi: 10.1038/tp.2017.186 (PMC5639239; doi:10.1038/tp.2017.186)
Supplement: Supplementary Table 4 [file tp2017186x5.docx]

| **Table S4.** ROI and exploratory whole brain (p < 0.001 uncorrected, k => 15) VBM analyses for sample 1. | | | | | | | |
| --- | --- | --- | --- | --- | --- | --- | --- |
| Contrast/Region | Side | Voxels | x | y | z | t | P |
| **Risk > No-Risk (ROI analysis)** |  |  |  |  |  |  |  |
| Superior medial frontal gyrus | R | 43 | 9 | 60 | 6 | 4.21 | 0.007 |
| Precentral gyrus | L | 17 | -20 | -22 | 64 | 3.88 | 0.033 |
| **Risk > No-Risk (exploratory analysis)** |  |  |  |  |  |  |  |
| Precentral gyrus | L | 25 | -18 | -22 | 64 | 3.98 | <0.001 |
| Middle frontal gyrus | L | 64 | -32 | -39 | 9 | 3.92 | <0.001 |
| Middle temporal gyrus | L | 101 | -56 | -12 | -18 | 3.53 | <0.001 |
| Superior temporal gyrus | L |  | -52 | -6 | -14 | 3.38 | <0.001 |
| Angular gyrus | R | 50 | 57 | -45 | 24 | 3.47 | <0.001 |
| **No-Risk > Risk (exploratory analysis)** |  |  |  |  |  |  |  |
| Planum polare | R | 135 | 40 | -4 | -21 | 4.36 | <0.001 |
| Risk group status was defined as carrying at least one risk allele (A allele). L: left; R: right; VBM: voxel-based morphometry; voxel: number of voxels per cluster; x, y, z: MNI coordinates | | | | | | | |
