# Supplementary material for: Modulation of defensive reactivity by GLRB allelic variation: converging evidence from an intermediate phenotype approach
Source: Transl Psychiatry. 2017 Sep 5;7(9):e1227–. doi: 10.1038/tp.2017.186 (PMC5639239; doi:10.1038/tp.2017.186)
Supplement: Supplementary Table 5 [file tp2017186x6.docx]

| **Table S5.** Statistical details for startle reflex habituation during ITIs divided by two time blocks (block1 & block2) for sample 1. | | | |
| --- | --- | --- | --- |
|  | df | t/F | p |
| **Repeated measures ANOVA** |  |  |  |
| Time | 1/103 | 30.723 | <0.001^1^ |
| *GLRB*-Risk | 1/103 | 3.328 | 0.071 |
| Time**GLRB*-Risk | 1/103 | 0.526 | 0.470 |
| Risk group status was defined as carrying at least one risk allele (A allele).  ITI: inter-trial interval. | | | |
