# Supplementary material for: Modulation of defensive reactivity by GLRB allelic variation: converging evidence from an intermediate phenotype approach
Source: Transl Psychiatry. 2017 Sep 5;7(9):e1227–. doi: 10.1038/tp.2017.186 (PMC5639239; doi:10.1038/tp.2017.186)
Supplement: Supplementary Table 6 [file tp2017186x7.docx]

| **Table S6**. Demographic and clinical characteristics of samples 1 and 2. Means (SD) except where noted. | | | | | | |
| --- | --- | --- | --- | --- | --- | --- |
|  | **sample 1** | | | | Chi^2^ or t (df) | p |
| fMRI fear conditioning sample (n = 48) | Combined Risk allele carriers (n = 20 [41.7%]) | | Combined No-Risk allele carriers (n = 28 [58.3%]) | |  |  |
| Female gender [n (%)] | 10 | (41.67) | 14 | (58.33) | <0.001 (1) | 1 |
| Years of education [n (%)] |  |  |  |  |  |  |
| 8 | 0 | (0.00) | 0 | (0.00) | n.a. | n.a. |
| 10 | 0 | (0.00) | 0 | (0.00) |  |  |
| 12-13 | 20 | (100.00) | 28 | (100.00) |  |  |
| NOS1ex1f s-carrier [n (%)] | 12 | (60.00) | 13 | (46.43) | 0.403 (1) | 0.526 |
| Age (years) | 23.55 | (2.06) | 25.93 | (3.52) | 2.706 (46) | 0.009 |
| US intensity (mA) | 6.05 | (3.96) | 5.84 | (3.68) | 0.191 (46) | 0.849 |
| ASI total | 14.55 | (5.90) | 14.11 | (7.14) | 0.227 (46) | 0.821 |
| Brain morphometry sample (n = 401) | Combined Risk allele carriers (n = 129 [32.2%]) | | Combined No-Risk allele carriers (n = 272 [67.8%]) | |  |  |
| Female gender [n (%)] | 70 | (25.74) | 41 | (31.78) | 1.311 (1) | 0.252 |
| Years of education^1^ [n (%)] |  |  |  |  |  |  |
| 8 | 0 | (0.00) | 2 | (0.74) | 1.388 (2) | 0.500 |
| 10 | 4 | (3.10) | 12 | (4.45) |  |  |
| 12-13 | 125 | (96.9) | 256 | (94.81) |  |  |
| Age^2^ (years) | 25.33 | (4.88) | 26.10 | (4.67) | 1.527 (396) | 0.128 |
| ASI total^2^ | 13.85 | (6.42) | 12.21 | (6.13) | 2.458 (398) | 0.014 |
| Affective startle sample  (n = 99) | Combined Risk allele carriers (n = 33 [33.3%]) | | Combined No-Risk allele carriers (n = 66 [66.6%]) | |  |  |
| Female gender^3^ [n (%)] | 17 | (53.16) | 37 | (56.06) | 0.003 (1) | 0.954 |
| Years of education^3^ [n (%)] |  |  |  |  |  |  |
| 8 | 1 | (3.03) | 0 | (0.00) | 2.386 (2) | 0.303 |
| 10 | 1 | (3.03) | 1 | (1.52) |  |  |
| 12-13 | 30 | (90.3) | 65 | (98.48) |  |  |
| Age (years)^3^ | 24.70 | (3.79) | 25.03 | (3.74) | 0.413 (96) | 0.681 |
| ASI total^3^ | 16.09 | (7.49) | 14.65 | (7.29) | 0.902 | 0.371 |
|  | **sample 2** | | | |  |  |

| fMRI fear conditioning sample (n = 36) | Combined Risk allele carriers (n = 24 [66.6%]) | | Combined No-Risk allele carriers ( n = 12 (33.3%]) | |  |  |
| --- | --- | --- | --- | --- | --- | --- |
| Female gender [n (%)] | 14 | (58.30) | 6 | (50.00) | 0.225 (1) | 0.635 |
| Years of education [n (%)] |  |  |  |  |  |  |
| 8 | 1 | (4.20) | 1 | (8.30) | 2.12 (2) | 0.346 |
| 10 | 7 | (29.20) | 1 | (8.30) |  |  |
| 12-13 | 16 | (66.70) | 10 | (83.30) |  |  |
| Site |  |  |  |  |  |  |
| Berlin | 7 | (29.20) | 6 | (50.00) | 1.800 (2) | 0.407 |
| Dresden | 9 | (37.50) | 4 | (33.30) |  |  |
| Münster | 8 | (33.30) | 2 | (16.70) |  |  |
| Age (years) | 35.25 | (9.08) | 41.50 | (12.67) | 1.703 (34) | 0.098 |
| Digit span forward | 7.88 | (1.90) | 8.50 | (2.39) | 0.854 (34) | 0.399 |
| Digit span backward | 7.17 | (2.22) | 6.17 | (1.90) | -1.33 (34) | 0.191 |
| TMT-A (sec) | 24.5 | (9.08) | 27.27 | (7.44) | 0.904 (34) | 0.373 |
| TMT-B (sec) | 55.63 | (16.23) | 61.83 | (22.06) | 0.865 (17) | 0.399 |
| US rating | 8.33 | (0.76) | 8.17 | (1.19) | -0.510 (34) | 0.613 |
| ASI total | 9.00 | (8.49) | 10.00 | (6.85) | 0.354 (34) | 0.726 |
| BDI II total | 1.46 | (2.09) | 1.67 | (1.83) | 0.294 (34) | 0.771 |

| Combined Risk group status was defined as carrying at least one risk allele in one out of four SNPs (rs 7688285: G/A with A allele as risk allele, rs17035763: G/A with A allele as risk allele, rs191260602: A/G with G allele as risk allele, and rs78726293: T/A with A allele as risk allele). ASI: Anxiety Sensitivity Index; BDI II: Beck Depression Inventory II; n.a.: not applicable; NOS1ex1f: *NOS1* ex1f-VNTR polymorphism; TMT-A: Trail Making Test A; TMT-B: Trail Making Test B; US: unconditioned stimulus (in mA). US rating: US subjective ratings (10 point Likert Scale); ^2^ n = 1 missing; ^3^ n = 1 missing. For sample 1, all subjects of the fear condition and brain morphology samples and 3 subjects of the affective startle sample were included in the GWAS analyses of the previous report ^4^. |
| --- |
