# Supplementary material for: Modulation of defensive reactivity by GLRB allelic variation: converging evidence from an intermediate phenotype approach
Source: Transl Psychiatry. 2017 Sep 5;7(9):e1227–. doi: 10.1038/tp.2017.186 (PMC5639239; doi:10.1038/tp.2017.186)
Supplement: Supplementary Table 7 [file tp2017186x8.docx]

| **Table S7**. Main effect of *GLRB* in the Combined *GLRB* Risk group from sample 1 on brain activation patterns during fear acquisition (ROI peak voxels are given). Small volume correction in pre-defined ROI analyses (FWE correction at p < 0.05) with a cluster forming threshold of p < 0.001. | | | | | | | | | |
| --- | --- | --- | --- | --- | --- | --- | --- | --- | --- |
| Contrast/Region | Side | Voxels | x | y | z | t | | | p |
| **Full acquisition: Risk > No-Risk** |  |  |  |  |  | | | | |
|  |  |  |  |  |  | |  |  | |
| Amygdala | L | 3 | -16 | -2 | -12 | | 4.64 | <0.001 | |
| Insula | R | 22 | 34 | -18 | 20 | | 4.61 | 0.007 | |
| Insula | R | 8 | 38 | -8 | 20 | | 4.18 | 0.024 | |
| **Full acquisition: No-Risk > Risk** |  |  |  |  | No differential activation | | | | |
|  |  |  |  |  |  | |  |  | |
| **Early acquisition: Risk > No-Risk** |  |  |  |  |  |  | | |  |
| Insula | L | 10 | -42 | 14 | 6 | 4.41 | | | 0.016 |
| Thalamus | R | 3 | 12 | -28 | 4 | 3.77 | | | 0.040 |
| Amygdala | L | 4 | -16 | -2 | -12 | 3.64 | | | 0.016 |
| **Early acquisition: No-Risk > Risk** | No differential activation | | | | | | | | |
| **Late acquisition: Risk > No-Risk** |  |  |  |  | No differential activation | | | | |
| Insula | R | 26 | 36 | -16 | 20 | 4.30 | | | 0.018 |
| Pons | L | 13 | -14 | -24 | -26 | 4.16 | | | 0.027 |
| **Late acquisition: No-Risk > Risk** | No differential activation | | | | | | | | |
| Combined Risk group status was defined as carrying at least one risk allele in one out of four SNPs (rs 7688285: G/A with A allele as risk allele, rs17035763: G/A with A allele as risk allele, rs191260602: A/G with G allele as risk allele, and rs78726293: T/A with A allele as risk allele). L: left; R: right; voxel: number of voxels per cluster; x, y, z: MNI coordinates. | | | | | | | | | |
