# Supplementary material for: Modulation of defensive reactivity by GLRB allelic variation: converging evidence from an intermediate phenotype approach
Source: Transl Psychiatry. 2017 Sep 5;7(9):e1227–. doi: 10.1038/tp.2017.186 (PMC5639239; doi:10.1038/tp.2017.186)
Supplement: Supplementary Table 8 [file tp2017186x9.docx]

| **Table S8.** Interaction effect *GLRB* and conditioned stimulus (CS+ vs. CS-) in the Combined *GLRB* Risk group from sample 1 on brain activation patterns during fear acquisition (ROI peak voxels are given). Small volume correction in pre-defined ROI analyses (FWE correction at p < 0.05) with a cluster forming threshold of p < 0.001. | | | | | | | | | |
| --- | --- | --- | --- | --- | --- | --- | --- | --- | --- |
| Contrast/Region | Side | Voxels | | | x | y | z | t | p |
| **Full acquisition: Risk > No-Risk (CS+ > CS-)** | | | No differential activation | | | | | | |
| Amygdala | L | 1 | | | -16 | -2 | -12 | 3.36 | 0.030 |
| **Full acquisition: Risk > No-Risk (CS- > CS+)** | | | | | | | | | |
| Amygdala | L | 1 | | |  | -4 | -20 | 3.44 | 0.024 |
| Hippocampus | L | 24 | | | -26 | -14 | -24 | 4.00 | 0.023 |
| Hippocampus | R | 21 | | | 40 | -26 | -14 | 4.05 | <0.001 |
| **Early acquisition: Risk > No-Risk (CS+ > CS-)** | | | | No differential activation | | | | | |
| **Early acquisition: Risk > No-Risk (CS- > CS+)** | | | | | | | | | |
| Thalamus | L | 10 | | | -6 | -12 | 12 | 3.76 | 0.047 |
| Anterior cingulate gyrus | L | 31 | | | 0 | 32 | 30 | 4.45 | 0.012 |
| **Late acquisition: Risk > No-Risk (CS+ > CS-)** | | | | No differential activation | | | | | |
| **Late acquisition: Risk > No-Risk (CS- > CS+)** | | | | | | |  | | |
| Hippocampus | R | 10 | | | 38 | -26 | -14 | 3.86 | 0.012 |
| Combined Risk group status was defined as carrying at least one risk allele in one out of four SNPs (rs 7688285: G/A with A allele as risk allele, rs17035763: G/A with A allele as risk allele, rs191260602: A/G with G allele as risk allele, and rs78726293: T/A with A allele as risk allele). L: left; R: right; voxel: number of voxels per cluster; x, y, z: MNI coordinates. | | | | | | | | | |
