# Supplementary material for: Modulation of defensive reactivity by GLRB allelic variation: converging evidence from an intermediate phenotype approach
Source: Transl Psychiatry. 2017 Sep 5;7(9):e1227–. doi: 10.1038/tp.2017.186 (PMC5639239; doi:10.1038/tp.2017.186)
Supplement: Supplementary Table 9 [file tp2017186x10.docx]

| **Table S9.** ROI and exploratory whole brain (p < 0.001 uncorrected, k => 15) VBM analysis for the Combined *GLRB* Risk group sample 1. | | | | | | | |
| --- | --- | --- | --- | --- | --- | --- | --- |
| Contrast/Region | Side | Voxels | x | y | z | t | p |
| **Risk > No-Risk (ROI analysis)** |  |  |  |  |  |  |  |
| Superior medial frontal gyrus | R | 64 | 9 | 60 | 6 | 4.30 | 0.005 |
| **Risk > No-Risk (exploratory analysis)** |  |  |  |  |  |  |  |
| Precentral gyrus | L | 22 | -6 | -22 | 70 | 3.96 | <0.001 |
| Posterior cingulate gyrus | R | 108 | 6 | -30 | 28 | 3.68 | <0.001 |
| Superior medial frontal gyrus | L | 36 | -8 | 62 | 4 | 3.67 | <0.001 |
| Angular gyrus | R | 19 | 57 | -51 | 27 | 3.60 | <0.001 |
| Inferior frontal gyrus | R | 55 | 51 | 45 | -0 | 3.58 | <0.001 |
| Temporal fusiform cortex anterior | L | 33 | -34 | -6 | -33 | 3.47 | <0.001 |
| Inferior occipital gyrus | L | 19 | -32 | -90 | -2 | 3.46 | <0.001 |
| Middle frontal gyrus | L | 16 | -28 | 36 | 34 | 3.32 | <0.001 |
| Middle occipital gyrus | R | 16 | 36 | -72 | 37 | 3.32 | <0.001 |
| Parahippocampal gyrus | L | 15 | -33 | -24 | -23 | 3.24 | 0.001 |
| **No-Risk > Risk (exploratory analysis)** |  |  |  |  |  |  |  |
| Planum polare | R | 132 | 39 | -3 | -20 | 4.23 | <0.001 |
| Occipital fusiform gyrus | R | 34 | 38 | -70 | -12 | 3.68 | <0.001 |
| Combined Risk group status was defined as carrying at least one risk allele in one out of four SNPs (rs 7688285: G/A with A allele as risk allele, rs17035763: G/A with A allele as risk allele, rs191260602: A/G with G allele as risk allele, and rs78726293: T/A with A allele as risk allele). L: left; R: right; VBM: voxel-based morphometry; voxel: number of voxels per cluster; x, y, z: MNI coordinates; Please note: due to differences in Combined Risk groups in mean ASI sum scores (see Table S4), ASI sum score was used as a covariate in the VBM group analysis (one missing value within the GLRB was imputed with group mean ASI sum score). | | | | | | | |
