# Supplementary material for: Modulation of defensive reactivity by GLRB allelic variation: converging evidence from an intermediate phenotype approach
Source: Transl Psychiatry. 2017 Sep 5;7(9):e1227–. doi: 10.1038/tp.2017.186 (PMC5639239; doi:10.1038/tp.2017.186)
Supplement: Supplementary Table 10 [file tp2017186x11.docx]

| **Table S10.** Main effect of *GLRB* in the Combined *GLRB* Risk group sample 2 on brain activation patterns during fear acquisition and extinction (cluster peak voxels are given). | | | | | | | |
| --- | --- | --- | --- | --- | --- | --- | --- |
| Contrast/Region | Side | Voxels | x | y | z | t | p ^1^ |
| **Overall: Risk > no-risk** |  |  |  |  |  |  |  |
| Insula | R | 728 | 34 | 14 | 14 | 3.87 | <0.001 |
| Postcentral gyrus | L | 343 | -40 | 18 | 28 | 3.41 | <0.001 |
| Inferior frontal operculum | L | 240 | -40 | 12 | 12 | 3.11 | 0.001 |
| **Overall: No-risk > risk** |  |  |  |  |  |  |  |
| Calcarine gyrus | L | 440 | -10 | -98 | -8 | 4.90 | <0.001 |
| Calcarine gyrus | R | 151 | 22 | -98 | 2 | 4.06 | <0.001 |
| Middle occipital gyrus | L | 180 | -30 | -94 | 10 | 3-83 | <0.001 |
| **Acquisition: risk > no-risk** |  |  |  |  |  |  |  |
| Insula | R | 681 | 32 | 14 | 14 | 3.79 | <0.001 |
| **Acquisition: no-risk < risk** |  |  |  |  |  |  |  |
| Lingual gyrus | L | 375 | -14 | -95 | -12 | 4.74 | <0.001 |
| **Extinction: risk > no-risk** |  |  |  |  |  |  |  |
| Precuneus (2.00 mm dev.) | R | 548 | 20 | -40 | 44 | 4.24 | <0.001 |
| Postcentral gyrus | L | 604 | -30 | -30 | 46 | 3.72 | <0.001 |
| Insula (5.00 mm dev.) | L | 251 | -26 | -18 | 18 | 3.18 | 0.001 |
| Insula | R | 189 | 36 | 6 | 14 | 3.07 | 0.001 |
| **Extinction: no-risk > risk** |  |  |  |  |  |  |  |
| Calcarine gyrus | L | 287 | -8 | -98 | -6 | 3.87 | <0.001 |
| Combined Risk group status was defined as carrying at least one risk allele in one out of four SNPs (rs 7688285: G/A with A allele as risk allele, rs17035763: G/A with A allele as risk allele, rs191260602: A/G with G allele as risk allele, and rs78726293: T/A with A allele as risk allele). L: left; R: right; voxel: number of voxels per cluster; x, y, z: MNI coordinates; dev. Deviation (in mm) from the identified anatomical structure using anatomic automatic labelling (aal). ^1^ p < 0.005 (uncorr.) with a minimum cluster size of 142 contiguous voxels, indicating to correct for multiple comparisons at p <0.05. Please note: due to trendwise differences in Combined Risk groups in mean age (see Table S6), age instead of BDI II was used as a covariate in the 2^nd^ level fMRI analysis. | | | | | | | |
