# Supplementary material for: Modulation of defensive reactivity by GLRB allelic variation: converging evidence from an intermediate phenotype approach
Source: Transl Psychiatry. 2017 Sep 5;7(9):e1227–. doi: 10.1038/tp.2017.186 (PMC5639239; doi:10.1038/tp.2017.186)
Supplement: Supplementary Table 11 [file tp2017186x12.docx]

| **Table S11.** Interaction effect of *GLRB* and CS in the Combined *GLRB* Risk group sample 2 on brain activation patterns during fear acquisition and extinction (cluster peak voxels are given). | | | | | | | | |
| --- | --- | --- | --- | --- | --- | --- | --- | --- |
| Contrast/Region | Side | Voxels | x | y | z | t | p ^1^ | |
| **Full acquisition: Risk > No-Risk (CS+ > CS-)** | | | | | | | | |
| Angular gyrus (4 mm dev.) | R | 768 | 34 | -56 | 32 | 3.88 | <0.001 | |
| Angular gyrus | L | 459 | -38 | -60 | 36 | 3.38 | <0.001 | |
| **Full acquisition: Risk > No-Risk (CS- > CS+)** No differential activation | | | | | | | | |
| **Early acquisition: Risk > no-risk (CS+ > CS-)** | | | | | | | | |
| Angular gyrus (5.66 mm dev.) | R | 510 | 32 | -56 | 32 | 4.09 | <0.001 | |
| **Early acquisition: Risk > no-risk (CS- > CS+)** | | | | | | | | |
| Postcentral gyrus | R | 208 | 28 | -46 | 74 | 4.65 | <0.001 | |
| Middle temporal gyrus | R | 353 | 60 | -2 | -22 | 4.25 | <0.001 | |
| Middle cingulate gyrus^2^ | R | 1580 | 4 | -20 | 44 | 4.04 | <0.001 | |
| Middle occipital gyrus | L | 202 | -40 | -80 | 22 | 3.96 | <0.001 | |
| Middle frontal gyrus | L | 252 | -34 | 46 | 32 | 3.80 | <0.001 | |
| Suppementary motor area* | R | 18 | 4 | -20 | 48 | 3.78 | 0.016 | |
| Amygdala^3^ | R | 276 | 24 | 6 | -18 | 3.58 | <0.001 | |
| Amygdala | L | 853 | -22 | -2 | -18 | 3.56 | <0.001 | |
| Supplementary motor area* | L | 5 | 0 | 18 | 48 | 3,51 | 0.035 | |
| Precuneus | R | 223 | 4 | -56 | 66 | 3.00 | 0.001 | |
| **Late acquisition: Risk > no-risk (CS+ > CS-)** | | | | | | | | |
| Middle temporal gyrus | R | 1324 | 54 | -68 | 4 | 5.57 | <0.001 | |
| Rolandic operculum | R | 2020 | 48 | -14 | 18 | 4.63 | <0.001 | |
| Posterior cingulate gyrus | L | 2100 | -8 | -40 | 28 | 4.35 | <0.001 | |
| Hippocampus* | R | 42 | 40 | -22 | -10 | 3.90 | 0.005 | |
| Anterior cingulate gyrus | R | 458 | 4 | 10 | 25 | 3.70 | <0.001 | |
| Anterior cingulate gyrus* | L | 26 | -4 | 18 | 22 | 3.61 | 0.023 | |
| Middle temporal gyrus | L | 662 | -54 | -50 | 14 | 3.27 | <0.001 | |
| Superior parietal gyrus | L | 421 | -26 | -64 | 46 | 3.57 | <0.001 | |
| Postcentral gyrus | R | 407 | -56 | -14 | 20 | 3.53 | <0.001 | |
| Inferior parietal gyrus | R | 164 | 58 | -28 | 54 | 3.37 | <0.001 | |
| Precentral gyrus | L | 535 | -34 | 0 | 44 | 3.18 | <0.001 | |
| Superior temporal gyrus | L | 225 | -46 | -8 | -14 | 2.95 | 0.002 | |
| **Late acquisition: Risk > no-risk (CS- > CS+)** No differential activation | | | | | | | | |
| **Full extinction: Risk > No-Risk (CS+ > CS-)** No differential activation | | | | | | | | |
| **Full extinction: Risk > No-Risk (CS- > CS+)** | | | | | | | | |
| Anterior cingulate gyrus* | R | 11 | 4 | 34 | 16 | 3.33 | 0.044 | |
| Combined Risk group status was defined as carrying at least one risk allele in one out of four SNPs (rs 7688285: G/A with A allele as risk allele, rs17035763: G/A with A allele as risk allele, rs191260602: A/G with G allele as risk allele, and rs78726293: T/A with A allele as risk allele). CS: conditioned stimulus; CS+: CS that is followed by an unconditioned stimulus (reinforcement rate: 50%; only unpaired CS+ were included in the analysis); CS-: CS that is never followed by the US; early acquisition/extinction: first half of the respective phase including eight trials per stimulus; late acquisition/extinction second half of the respective phase (including eight trials per stimulus); L: left; R: right; voxel: number of voxels per cluster; x, y, z: MNI coordinates; dev.: deviation (in mm) from the identified anatomical structure using anatomic automatic labelling (aal). Please note: due to trendwise differences in Combined Risk groups in mean age (see Table S5), age instead of BDI II was used as a covariate in the 2^nd^ level fMRI analysis.  ^2^ Cluster including the right anterior cingulate gyrus; ^3^cluster including right insula; ^1^ p < 0.005 (uncorr.) with a minimum cluster size of 142 contiguous voxels, indicating to correct for multiple comparisons at p <0.05. * Small volume correction using aal masks (FWE correction at p < 0.05) with a cluster forming threshold of p < 0.001. No significant clusters were detected for the early or late extinction phase. | | | | | | | |  |
