# Supplementary material for: Modulation of defensive reactivity by GLRB allelic variation: converging evidence from an intermediate phenotype approach
Source: Transl Psychiatry. 2017 Sep 5;7(9):e1227–. doi: 10.1038/tp.2017.186 (PMC5639239; doi:10.1038/tp.2017.186)
Supplement: Supplementary Table 12 [file tp2017186x13.docx]

| **Table S12.** Statistical details for startle reflex habituation during ITIs divided by two time blocks (block1 & block2) for sample 1. | | | |
| --- | --- | --- | --- |
|  | df | t/F | p |
| **Repeated measures ANOVA** |  |  |  |
| Time | 1/97 | 46.191 | <0.001 |
| *GLRB*-Risk | 1/97 | 1.478 | 0.227 |
| Time**GLRB*-Risk | 1/97 | 1.529 | 0.219 |
| Combined Risk group status was defined as carrying at least one risk allele in one out of four SNPs (rs 7688285: G/A with A allele as risk allele, rs17035763: G/A with A allele as risk allele, rs191260602: A/G with G allele as risk allele, and rs78726293: T/A with A allele as risk allele). | | | |
