# Supplementary figures and images for: Modulation of defensive reactivity by GLRB allelic variation: converging evidence from an intermediate phenotype approach
Source: Transl Psychiatry. 2017 Sep 5;7(9):e1227–. doi: 10.1038/tp.2017.186 (PMC5639239; doi:10.1038/tp.2017.186)

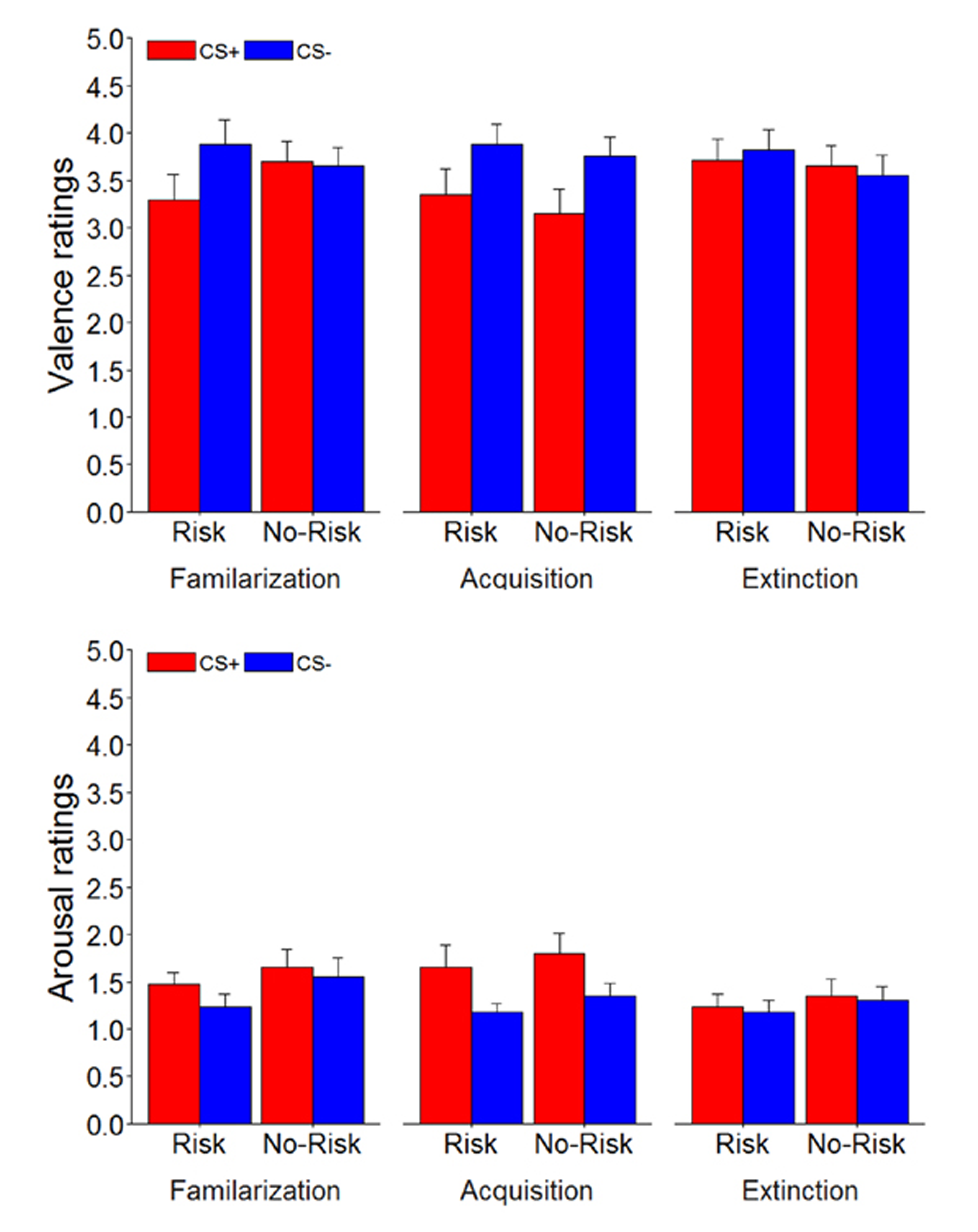

Supplement: Supplementary Figure 2 [file tp2017186x15.tif]

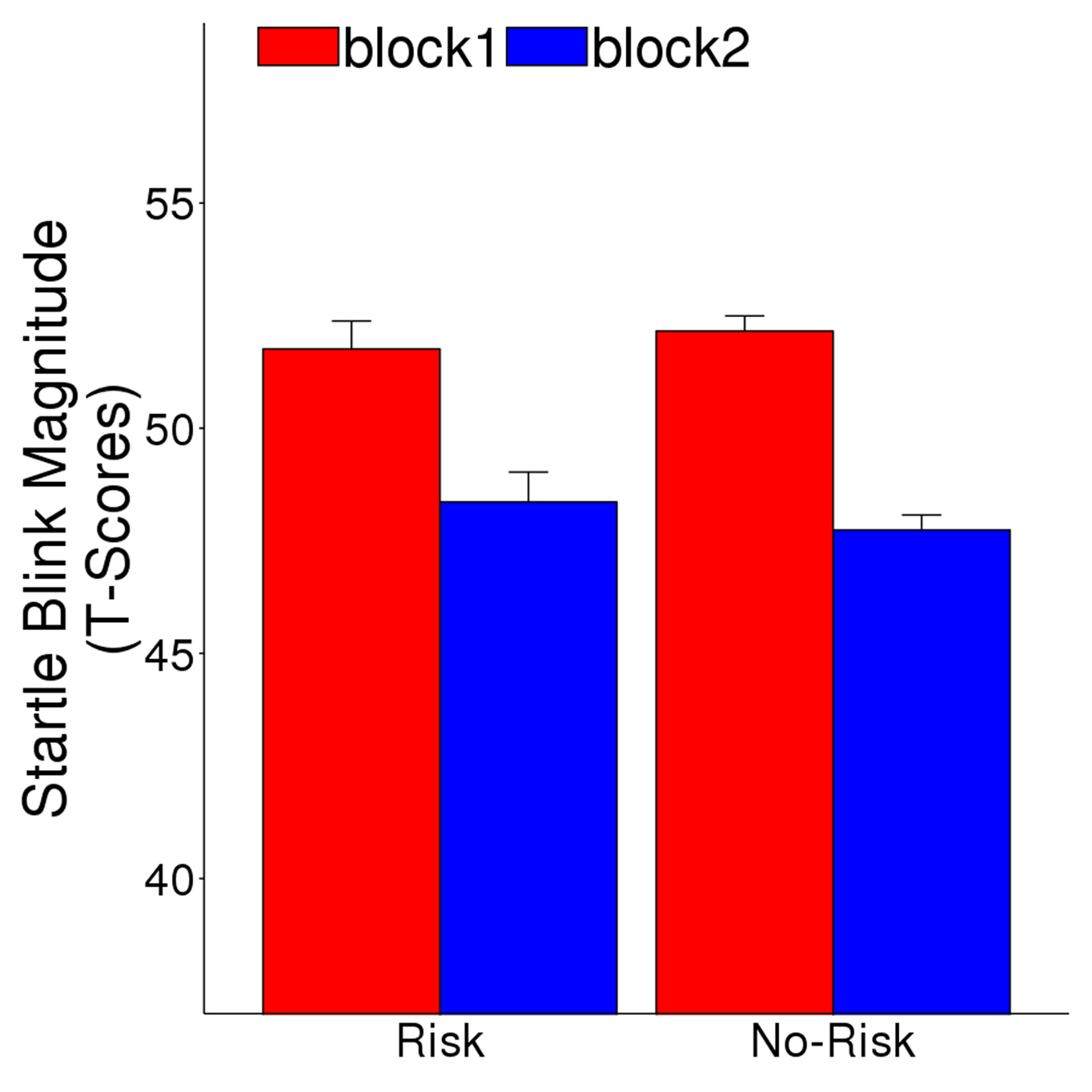

Supplement: Supplementary Figure 3 [file tp2017186x16.tif]

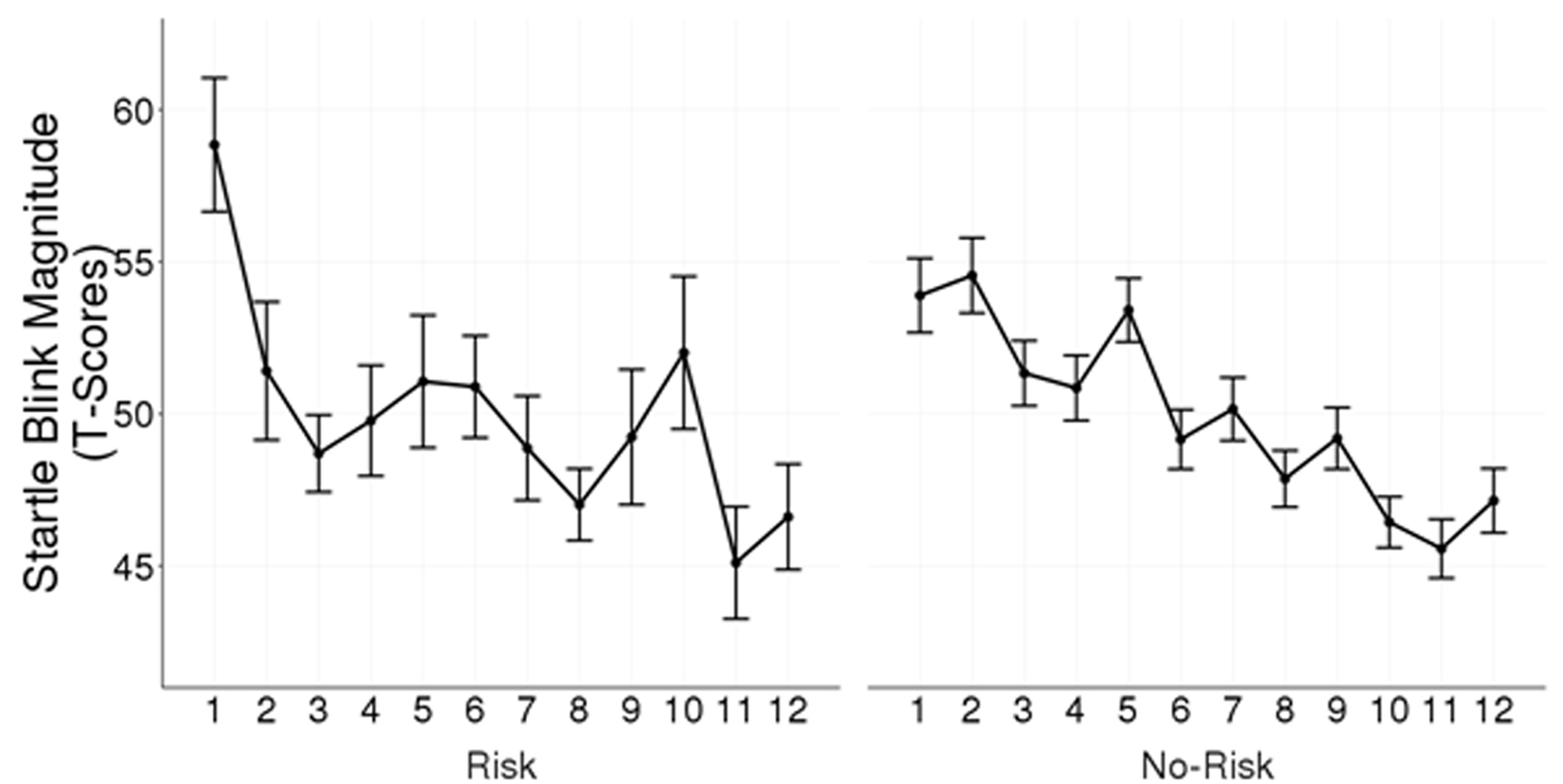

Supplement: Supplementary Figure 4 [file tp2017186x17.tif]
